# Microbiota-derived indole acetic acid extends lifespan through the AhR-Sirt2 pathway in *Drosophila*

**DOI:** 10.1128/msystems.01665-24

**Published:** 2025-04-08

**Authors:** Zheng Cao, Cui Zhang, Lijun Liu, Hehua Lei, Huabao Zhang, Yanmeng He, Xinzhi Li, Qingwei Xiang, Yu-Feng Wang, Limin Zhang, Gang Chen

**Affiliations:** 1 State Key Laboratory of Magnetic Resonance and Atomic and Molecular Physics, National Centre for Magnetic Resonance in Wuhan, Innovation Academy of Precision Measurement Science and Technology, Chinese Academy of Sciences (CAS), Wuhan, China; 2 University of Chinese Academy of Sciences, Beijing, China; 3 School of Pharmacy, Faculty of Medicine, Laboratory for Drug Discovery from Natural Resource, State Key Laboratory of Quality Research in Chinese Medicine, Macau University of Science and Technology, Macao, China; 4 Hubei Shizhen Laboratory, Department of Geriatrics, Hubei Provincial Hospital of Traditional Chinese Medicine, Hubei University of Chinese Medicine, Wuhan, China; 5 School of Life Sciences, Key Laboratory of Pesticide & Chemical Biology of Ministry of Education, Hubei Key Laboratory of Genetic Regulation and Integrative Biology, Central China Normal University, Wuhan, China; Vanderbilt University Medical Center, Nashville, Tennessee, USA

**Keywords:** Aging, AhR, Sirt2, *Drosophila*

## Abstract

Disruption of aryl hydrocarbon receptor (AhR) signaling and aberrant tryptophan metabolism have been shown to be highly associated with aging and age-related disorders. However, the underlying molecular mechanisms by which the AhR-mediated signaling pathway contributes to the aging process remain largely unknown. In this study, we find that aged *Drosophila* exhibits markedly reduced tryptophan metabolism leading to impaired AhR ligands, especially indole acetic acid (IAA), compared with their young controls. Supplementation with IAA, produced from *Lactobacillus* spp., dose-dependently extends the lifespan of *Drosophila* and improves healthy aging with resistance to starvation and oxidative stress. Mechanistically, activation of AhR by IAA markedly enhances *Sirt2* activity by binding to its promoter, thereby inhibiting downstream TOR signaling and related fatty acid and amino acid metabolism. Both *Ahr* and *Sirt2* mutant flies with IAA supplementation display a negligible lifespan extension, suggesting that AhR-mediated Sirt2 signaling contributes to lifespan extension in flies upon IAA supplementation. From the perspective of host metabolism, IAA supplementation significantly increases unsaturated fatty acids (UFAs) in aged flies, which are regarded to be beneficial for healthy status. These findings provide new insights into the physiological functions of AhR involved in the aging process by mediating Sirt2 signaling.

**IMPORTANCE:**

Disruption of aryl hydrocarbon receptor (AhR) signaling and aberrant tryptophan metabolism contribute to aging and age-related disorders, but the underlying molecular mechanisms are largely unknown. Using multiomics analyses combined with biochemical assays, this study reveals that AhR activation by indole acetic acid (IAA) effectively extends the lifespan accompanied by improved healthy aging in *Drosophila* via the AhR-Sirt2 pathway.

## INTRODUCTION

Aging is the progressive deterioration in the functions of tissues and organs directly contributing to healthspan, lifespan, and disease (1). It is a complicated physiological and pathological process that is regulated by many factors such as genes, the environment, and lifestyle (2, 3). Various mechanisms, including systemic inflammation and oxidative stress, dysregulated autophagy and immune senescence, dysbacteriosis, and host metabolic disorders, have been proposed for aging research (4). During the past several decades, aging research has transitioned from identifying host aging phenotypes to exploring the relevant genetic pathways (5). For example, circadian clocks, mitochondria and oxidative stress, and sirtuin deacetylases and several nutrient-sensing pathways, such as insulin/insulin-like growth factor (IGF) pathway, mechanistic target of rapamycin (mTOR), adenosine monophosphate-activated protein kinase (AMPK), have been identified as regulators of aging in animal models (6–8). Furthermore, various interventions including genetic, exercise, dietary restriction, and pharmacological approaches (rapamycin, metformin, nicotinamide mononucleotide, senolytics, etc.) have shown potential to increase healthspan and/or lifespan (9–12).

The aryl hydrocarbon receptor (AhR) is a cytoplasmic transcription factor that is known to have a pivotal role in mediating many physiological processes such as cell proliferation and regeneration, immune response, and intestinal homeostasis as well as the well-established role in xenobiotic detoxification (13, 14). Over the past several decades, numerous xenobiotics with high-affinity binding to AhR such as dioxins, dibenzofurans, and biphenyls were identified to activate AhR resulting in a series of toxicological outcomes and even tumorigenesis in rodents and humans (15, 16). More recently, many lower-affinity agonists of AhR have been identified from commercial and consumer products, fruits, and vegetables to exhibit a wide variety of physiological functions in different metabolic diseases. Of particular note is the presence of endogenous AhR ligands at the site of the intestinal epithelial barrier that contributes to the gut microflora and host immunity (17–19). In the gastrointestinal tract, tryptophan metabolism via the gut bacteria from dietary sources produces indole and its derivatives such as indole-3-aldehyde (IAld), indole acetic acid (IAA), indole-3-propionic acid (IPA), indole lactic acid (ILA), indoleacrylic acid (IA), and indole-3-acetaldehyde (IAAld) acting as endogenous ligands of AhR that are capable of enhancing host immunity and metabolic hemostasis (20). Previous pre-clinical and clinical studies showed that the decreased ability of the microbiota to produce AhR ligands led to impaired gut barrier functions and AhR agonist activity, ultimately facilitating the development of more severe metabolic syndrome such as high blood pressure, diabetes, and obesity (18). Disruption of AhR signaling and aberrant tryptophan metabolism have also been shown to be highly associated with age-related disorders such as neurodegenerative and cardiovascular diseases (21). A recent study suggested that indoles from commensal microbiota can extend the healthspan of diverse organisms including *C. elegans*, *Drosophila melanogaster*, and mice rather than their lifespan, which warrants further investigation (22). A comparative cohort study of wild-type and AhR-null mice along aging showed that AhR deficiency induced marked prematurely aged phenotypes accompanied by early inflammation, impaired spatial memory and blood glucose homeostasis, and collapse of the immune system (23). Based on this close relationship between AhR and longevity, we hypothesized that activation of AhR by endogenous ligands may be beneficial for longevity.

Sirtuins (SIRTs) are evolutionarily conserved nicotinamide adenine dinucleotide (NAD+)-dependent protein deacetylases that regulate numerous metabolic pathways and participate in aging-related diseases (24, 25). Among the SIRTs, SIRT2 is a histone deacetylase that predominantly resides in the cytosol during most cell cycles, while it is expressed in the nucleus and functionally associated with chromatin during G2/M transition and mitosis. SIRT2 catalyzes deacetylation of histone H4K16 aiding in DNA replication and repair (26). Furthermore, SIRT2 modulators have been demonstrated to inhibit lipopolysaccharide-stimulated production of TNF-α, thereby suppressing neuroinflammation (27, 28). A previous study revealed that *Sirt2* knockout significantly exaggerated cardiac hypertrophy and fibrosis in elderly mice. Conversely, SIRT2 overexpression can effectively ameliorate aging-related cardiac hypertrophy by activating the liver kinase B1 (LKB1)-AMPK pathway (29). Moreover, overexpression of SIRT2 or supplementation with NAD +precursor nicotinamide mononucleotide (NMN) markedly increases the lifespan of mice via induction of the mitotic checkpoint kinase BubR1 (30). Collectively, these findings indicate that both AhR and SIRT2 are more or less involved in the aging process and aging-related diseases. However, the relationship between AhR and SIRT2-linked signaling in the aging process remains elusive.

In this study, aged *Drosophila* exhibited markedly reduced tryptophan metabolism and indole derivatives, especially indole acetic acid (IAA), compared with young controls. Supplementing with IAA extended the lifespan of *Drosophila* via activation of AhR accompanied by resistance to starvation, oxidative stress, and restoration of gut barrier function during aging. Mechanistically, activation of AhR markedly elevated the protein level of SIRT2 by binding to the promoter region of the *Sirt2* gene. Subsequently, phosphorylation of the TOR protein and related downstream metabolic pathways, including cellular lipid, amino acid, and glucan catabolic processes, were inhibited in *Drosophila* during aging.

## RESULTS

### Altered gut microbiota composition and reduced AhR agonists in *Drosophila* during aging

**Fig 1:**
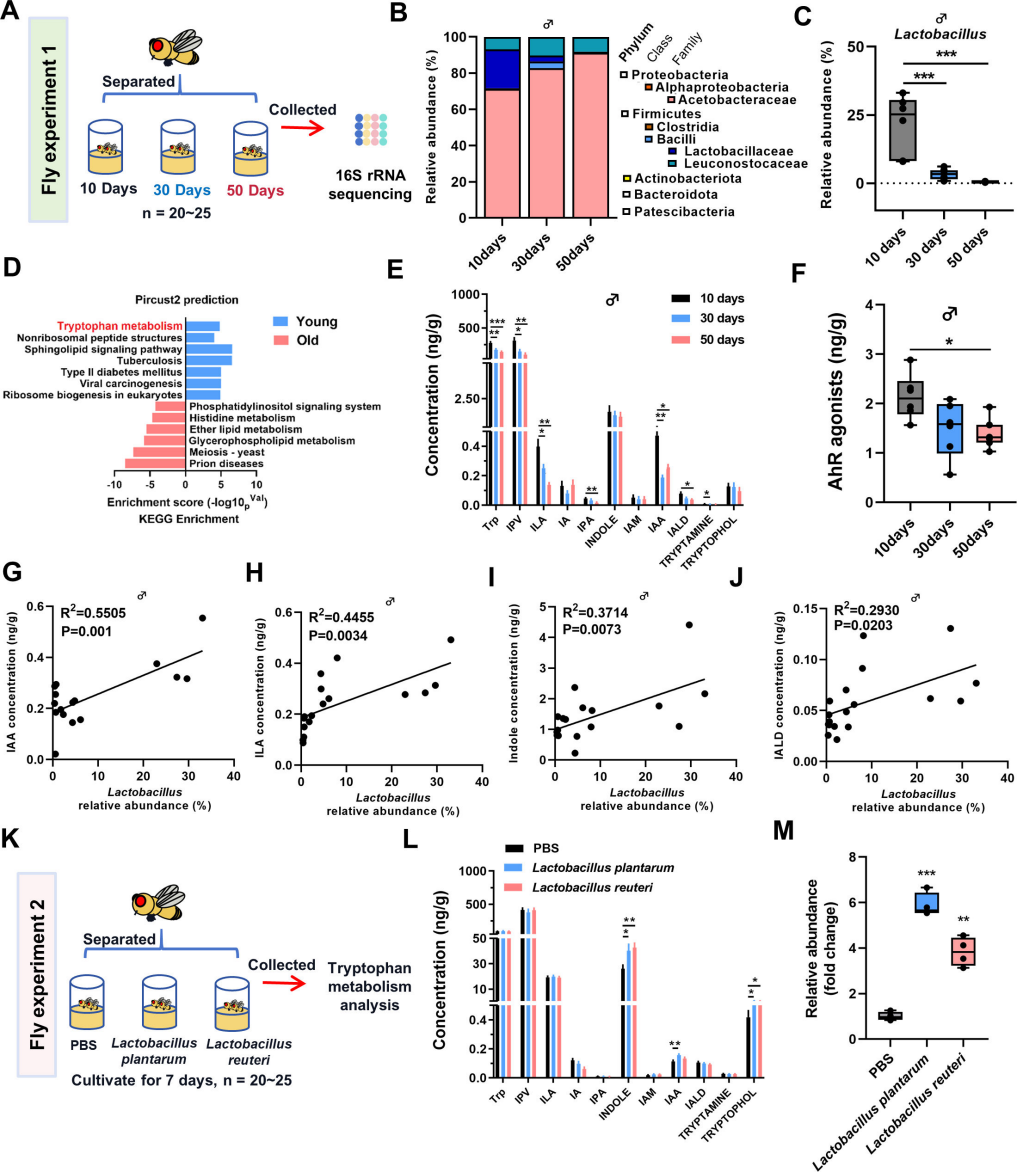
Altered microbiota composition and reduced AhR agonists during aging in *Drosophila*. (A) Schematic illustration of the fly experiment 1. (B) Changes in gut microbiota in 10-, 30-, and 50-day-old male *Drosophila* (*n* = 6–8 biological duplicates, 10 male flies for each duplicate). (C) Changes in *Lactobacillus* abundance in 10-, 30-, and 50-day-old male *Drosophila* (*n* = 6–8 biological duplicates, 10 male flies for each duplicate). *P* values correspond to one-way ANOVA. (D) Pircust2 analysis of gut microbiota change in 10-day (Young) and 50-day (Old) male *Drosophila* (*n* = 6–8 biological duplicates, 10 male flies for each duplicate). (E) Metabolites involved in the indole metabolism pathway changes in *Drosophila* at 10, 30, and 50 days old (*n* = 8 biological duplicates, 10 male flies for each duplicate). Data are shown as mean ± SEM. *P* values correspond to one-way ANOVA. (F) Changes in AhR ligand content in *Drosophila* at 10, 30, and 50 days old (*n* = 8 biological duplicates, 10 male flies for each duplicate). *P* values correspond to one-way ANOVA. (G–J) Correlation between *Lactobacillus* abundance and AhR agonist concentration. The correlation coefficient (R^2^) and *P*-values from Pearson correlation analysis are shown. (K) Schematic illustration of the fly experiment 2. (L) Metabolites involved in Indole metabolism pathway changes in *Drosophila* after *Lactobacillus* colonization (*n* = 4 biological duplicates, 10 male flies for each duplicate). *P* values correspond to one-way ANOVA. (M) Changes in the content of *Lactobacillus spp*. in the intestinal tract of *Drosophila* after colonization by *L. plantarum* and *L. reuteru* (*n* = 4 biological duplicates, 10 male flies for each duplicate). Data are shown as mean ± SEM. *P* values correspond to one-way ANOVA. *P < 0.05, **P < 0.01 and ***P < 0.001.

16S rRNA gene sequencing was performed to investigate the changes of gut microbiota in *Drosophila* at different ages (10, 30, and 50 days) (Fig. 1A). The results showed that relative abundance of Proteobacteria and Acetobacteraceae was markedly upregulated together with significant downregulation of Firmicutes and Lactobacillaceae in both male and female *Drosophila* during aging (Fig. 1B; Fig. S1A). Notably, a significant decrease in the relative abundance of the genera *Lactobacillus* was also observed in *Drosophila* during aging (Fig. 1C; Fig. S1B). Pircust2 prediction of gut microbiota revealed that aged *Drosophila* exhibited markedly reduced tryptophan metabolism compared with young controls (Fig. 1D). Targeted metabolomic analysis showed that the levels of indole metabolites such as ILA, IAA, and IALD were significantly decreased in *Drosophila* during aging (Fig. 1E; Fig. S2A). Apart from indole metabolites, significantly altered tryptophan metabolites involved in kynurenine (Kyn) and serotonin (5-HT) pathways were also observed in *Drosophila* during aging (Fig. S2B through E). Correspondingly, the abundance of these specific microbiota metabolites known as AhR agonists presented a marked decline in both male and female *Drosophila* during aging (Fig. 1F; Fig. S2F).

The relative abundance of *Lactobacillus* was positively correlated with the concentration of indole metabolites, including IAA, ILA, indole, and IALD in *Drosophila* during aging (Fig. 1G through J; Fig. S2G through K). To further verify the relationship between *Lactobacillus* and IAA, we colonized *Drosophila* with two *Lactobacillus* species and quantitatively measured the levels of indole metabolites (Fig. 1K) (31). The results showed a significant increase in *Lactobacillus* abundance in the intestine of *Drosophila* after colonization with *Lactobacillus plantarum* and *Lactobacillus reuteru* (Fig. 1L). Targeted analysis of tryptophan metabolism revealed that both *Lactobacillus plantarum* and *Lactobacillus reuteru* increased the level of indole and tryptophol, while *Lactobacillus plantarum* treatment increased IAA level (Fig. 1M). Moreover, we obtained germ-free flies by ABX treatment and measured the levels of IAA. The results showed a significant decrease in IAA compared to wild-type *Drosophila*. After colonizing *Lactobacillus plantarum*, the levels of *Lactobacillus* and IAA were increased (Fig. S3A through C), suggesting that IAA is derived from *Lactobacillus*.

### Supplementation with IAA extends lifespan in *Drosophila* via AhR

**Fig 2:**
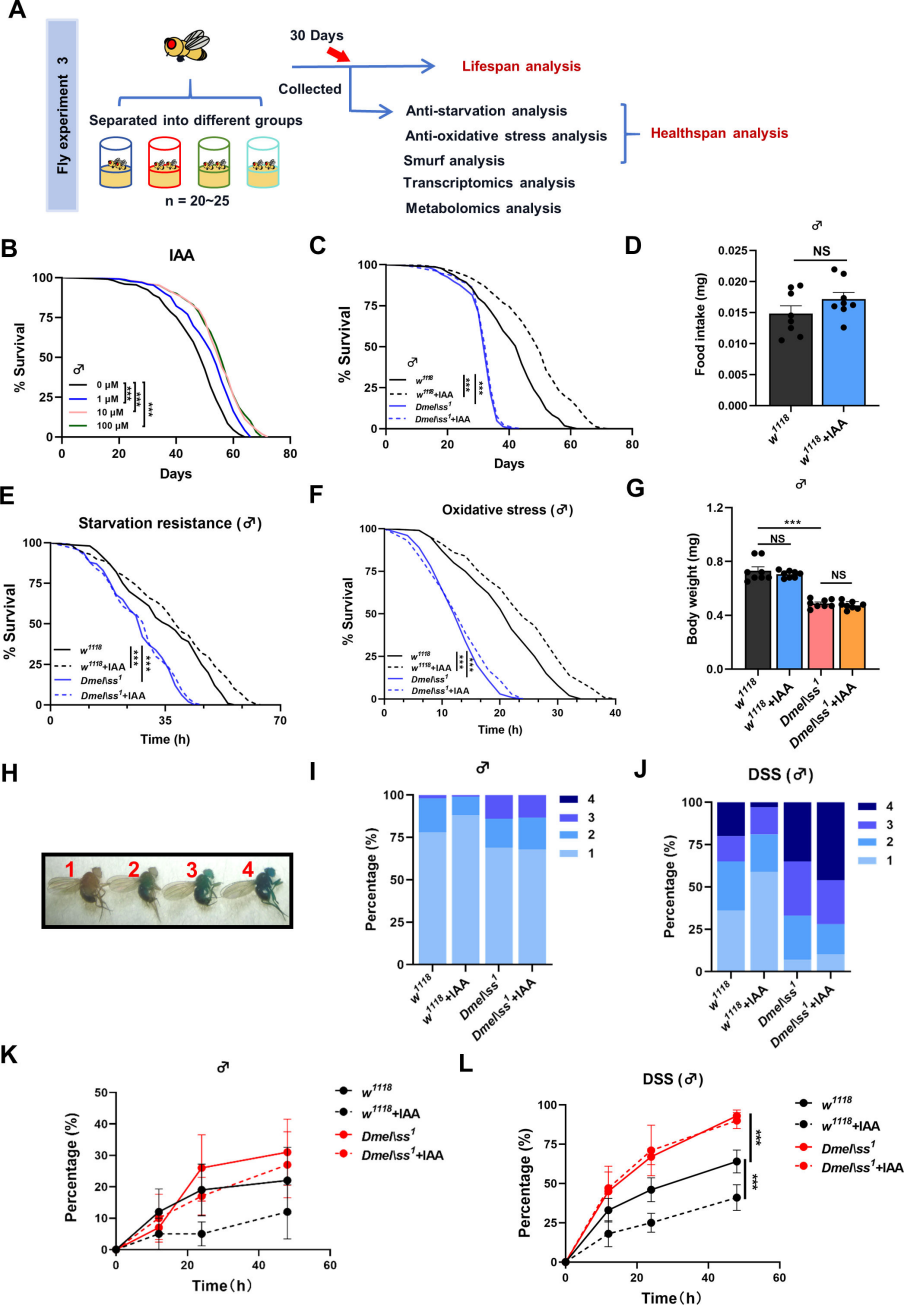
Supplement of IAA extends lifespan and improves healthspan in *Drosophila*. (A) Schematic illustration of the fly experiment 3. (B) Kaplan–Meier survival curves of supplement IAA with different concentrations in *w^1118^* flies (*n* = 5 biological duplicates, 40–45 male flies for each duplicate). (C) Kaplan–Meier survival curves of supplement with 50 µM IAA in *w^1118^* and *AhR* mutant flies (*n* = 5 biological duplicates, 40–45 male flies for each duplicate). (D) Food intake assay of supplement with 50 µM IAA in *w^1118^* flies (*n* = 8 biological duplicates, 10 male flies for each duplicate). *P* values correspond to unpaired t-test. (E) Starvation tolerance of supplement with 50 µM IAA in *w^1118^* and *AhR* mutant flies (*n* = 8 biological duplicates, 20–25 male flies for each duplicate). (F) Survival rate under oxidative stress (5% H_2_O_2_) of supplement with 50 µM IAA in *w^1118^* and *AhR* mutant flies (*n* = 8 biological duplicates, 20–25 male flies for each duplicate). (G) Body weight of 30-day-old *w^1118^* and *AhR* mutant flies (*n* = 8 biological duplicates, 10 male flies for each duplicate). *P* values correspond to one-way ANOVA. (H) Smurf rating (*n* = 4 biological duplicates, 20–25 male flies for each duplicate). (I, K) Smurf rating of 30-day male flies in *w^1118^* and *AhR* mutant flies of supplement with 50 µM IAA (*n* = 4 biological duplicates, 20–25 male flies for each duplicate). (J, L) Smurf rating of 30-day male flies after 3% DSS treatment in *w^1118^* and *AhR* mutant flies of supplement with 50 µM IAA (*n* = 4 biological duplicates, 20–25 male flies for each duplicate). Panels K and L drawn with the cumulated proportion of flies with a smurf rating of 2, 3, and 4 (level ≥2). Data are shown as mean ± SEM. *P* values correspond to two-way ANOVA (or mixed model). Kaplan–Meier lifespan curves were analyzed by log-rank (Mantel–Cox) test, *P < 0.05, **P < 0.01, and ***P < 0.001.

Given that these microbiota-derived AhR ligands were closely associated with aging in *Drosophila*, we next investigated whether treatment of AhR ligands with selection of IAA at different dosages can impact lifespan (Fig. 2A). Supplementation with IAA (0, 1, 10, and 100 µM) markedly extended lifespan in a dose-dependent manner in *w^1118^ Drosophila* (Fig. 2B; Fig. S4A). Compared with wild-type *Drosophila*, *Ahr* mutant *Drosophila* (*Dmel\ss^1^*) exhibited a marked decrease in lifespan, with the flies surviving under our culture conditions for less than 45 days (Fig. 2C; Fig. S4B). IAA supplementation (50 µM) extended the lifespan by approximately 15% (about 10 days) in *w^1118^ Drosophila* (Table S1), whereas no significant changes were observed in the lifespan of *Ahr* mutant *Drosophila* with and without IAA treatment (Fig. 2C; Fig. S4B). We also found that IAA supplementation did not significantly disturb food intake (Fig. 2D; Fig. S4C) and body weight (Fig. 2G; Fig. S4F) in *w^1118^ Drosophila* with both genders.

To determine whether AhR and IAA supplementation impact healthspan in *Drosophila*, 30-day wild-type and *Ahr* mutant flies with and without IAA treatment were harvested to examine their starvation resistance, oxidative stress, and gut vulnerability. Clearly, wild-type aged flies exhibited much stronger capability in resisting starvation (Fig. 2E; Fig. S4D) and oxidative stress than *Ahr* mutant flies (Fig. 2F; Fig. S4E). Notably, IAA supplementation induced a significant boost in the resilience against starvation (Fig. 2E; Fig. S4D) and oxidative stress (Fig. 2F; Fig. S4E) in wild-type aged flies rather than in *Ahr* mutant ones. Using Smurf assays (Fig. 2H; Fig. S4G) indicating the Brilliant Blue dye leaking from the gut, we found that the *Ahr* mutant induced a noticeable surge in gut vulnerability in flies (Fig. 2I; Fig. S4H). IAA treatment markedly reduced the gut permeability in wild-type flies rather than *Ahr* mutant flies (Fig. 2K; Fig. S4J). Following exposure to 3% DSS, *w^1118^* flies exhibited a significant disruption of the intestinal barrier that was expectedly improved by IAA supplementation (Fig. 2J; Fig. S4I). Compared with *w^1118^* flies, *Ahr* mutant flies showed a noticeable destruction of intestinal barrier integrity that was not significantly improved by IAA supplementation (Fig. 2L; Fig. S4K).

### Supplementation with IAA promotes *Sirt2* expression

**Fig 3:**
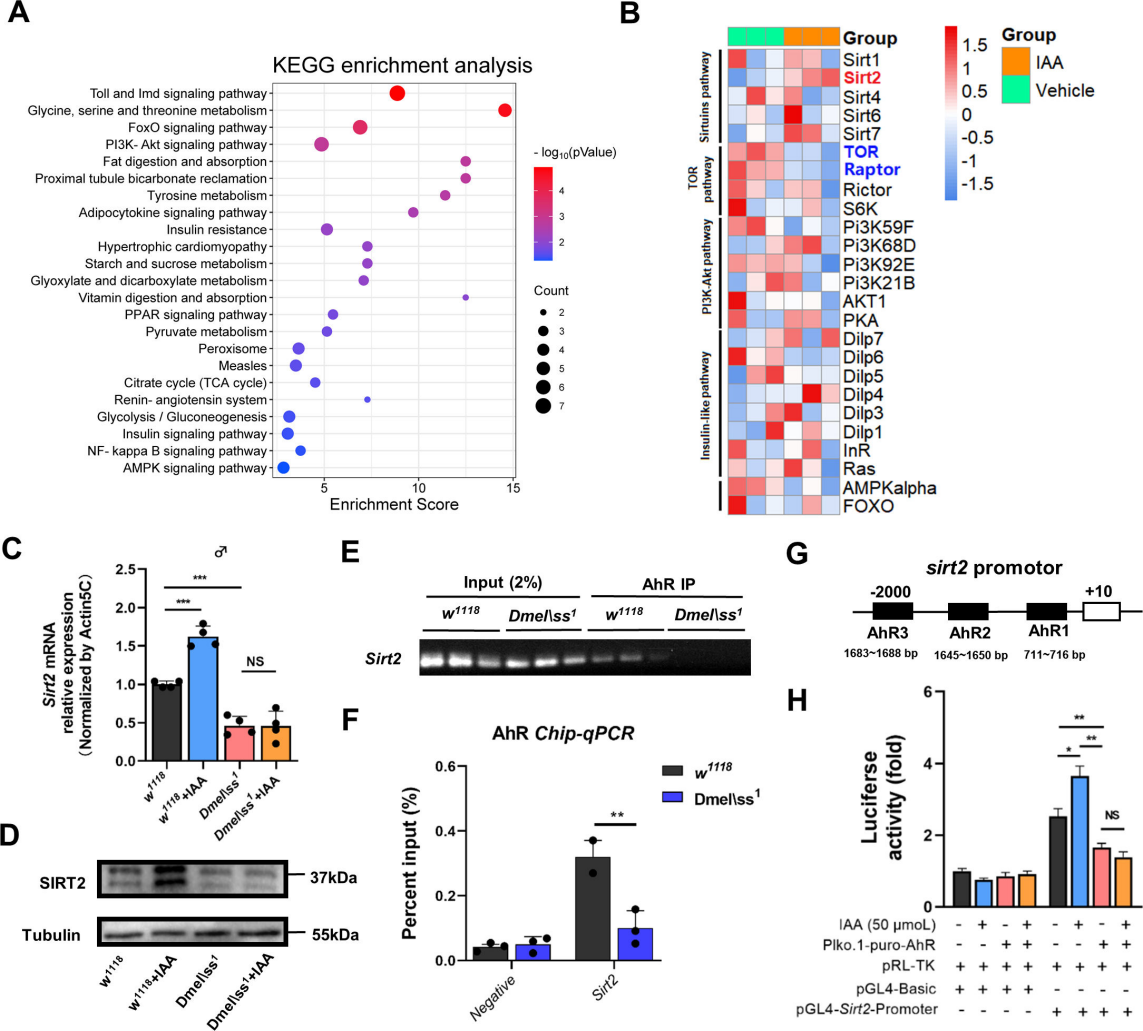
Transcriptomic analysis showed that IAA supplementation extends *Drosophila* lifespan by activating the Sirt2 pathway. (A) KEGG enrichment analysis screened top upregulated or downregulated pathways signaling pathways related to aging (*n* = 3 biological duplicates, 50 male flies for each duplicate). (B) Clustering heatmap of aging-related gene expression plotted by log10(TPM +1). (C) qPCR analysis of Sirt2 mRNA expression in male 30-day-old *w^1118^* and AhR mutant flies treated with IAA (*n* = 4 biological duplicates, 25 male flies for each duplicate). *P* values correspond to one-way ANOVA. (D) Western blot and protein quantification of SIRT2 (*n* = 3 biological duplicates, 25 male flies for each duplicate). (E) DNA electrophoretic gels showed the binding site of the Sirt2 promoter interacting with AhR (*n* = 3 biological duplicates, 30 male flies for each duplicate). (F) Chip-qpcr showed the binding site of the Sirt2 promoter interacting with AhR (*n* = 3 biological duplicates, 30 male flies for each duplicate). *P* values correspond to one-way ANOVA. (G) Schematic of Sirt2 gene promoter construct of AhR gene. The JASPAR database predicted that AhR-binding sites are present spanning from −1,683 to −1,688 bp, −1,645 to −1,650 bp, and −711 to −716 bp. (H) Dual luciferase assay in 293T cells with negative control (NC) and AhR gene knockdown (shAhR) stimulated by IAA (*n* = 4 biological duplicates). Data are shown as mean ± SEM. *P* values correspond to one-way ANOVA. *P < 0.05, **P < 0.01 and ***P < 0.001.

Next, we explored the underlying mechanisms of how AhR activation by IAA supplementation improves lifespan and healthspan in *Drosophila*. Transcriptomics analysis of aged flies showed that IAA supplementation enriched many inflammation-related pathways (Toll-like and NF-kappa B) and aging-related signaling pathways such as FOXO, PI3K-Akt, and insulin resistance, amino acid, and fatty acid metabolism (Fig. 3A). Notably, IAA supplementation induced marked upregulation in the mRNA level of deacetylase *Sirt2* and downregulation of rapamycin target protein (TOR) and TORC1 complex protein Raptor in aged flies (Fig. 3B). Interestingly, in comparison with wild-type flies, *Ahr* mutant *Drosophila* exhibited much lower *Sirt2* expression at both mRNA and protein levels that were markedly upregulated in *w^1118^* flies following IAA supplementation rather than in *Ahr* mutant *Drosophila* (Fig. 3C and D).

To further investigate the potential *Sirt2* gene targets of AhR, genomic DNA fragments of *Sirt2* bound by AhR were simultaneously analyzed by ChIP assays. Immunoprecipitated DNA was amplified and quantified by qPCR with primers for the *Sirt2* promoter. The results showed that the PCR product for the *Sirt2* promoter exhibited a marked downregulation when viewed on an agarose gel, and the percentage of *Sirt2* promoter in AhR-recognized genomic DNA fragments decreased significantly after *Ahr* mutation in *Drosophila* (Fig. 3E and F). Importantly, we discovered that at least three binding sites of the *Sirt2* promoter from −2,000 bp to +10 bp interact with AhR protein via simulation of the JASPAR database (Fig. 3G). To experimentally validate AhR binding to the promoter of *Sirt2*, we generated a homologous reporter construct comprising the −2,000/+10 bp *Sirt2* promoter cloned upstream of luciferase. After co-transfection of this reporter construct into HEK 293T cells with and without *Ahr*-knockdown, the luciferase activity in *Ahr*-knockdown cells is lower than that in wild-type cells. Of note, IAA supplementation induced a significant elevation in *Sirt2* reporter activity, whereas no significant change was observed in *Ahr*-knockdown cells upon IAA supplementation (Fig. 3H).

### Activation of AhR regulates aging-related metabolic pathways in *Drosophila*

**Fig 4:**
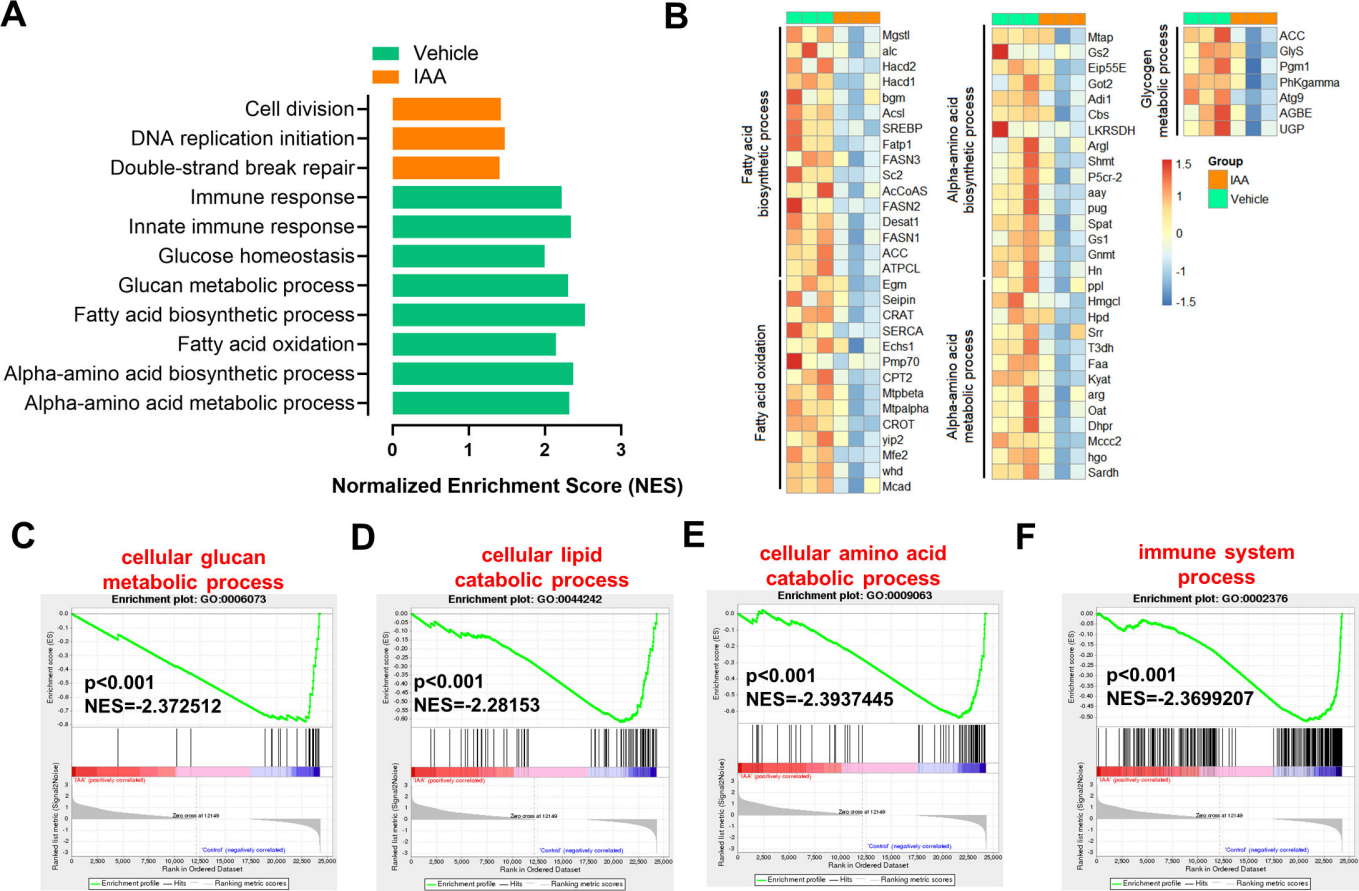
Activation of AhR regulates aging-related metabolic pathways in *Drosophila*. (A) Gene set enrichment analysis (GSEA) analysis showed top upregulated or downregulated pathways signaling pathways in flies treated with IAA in 30-day-old *Drosophil*a (*n* = 3 biological duplicates, 50 male flies for each duplicate). (B) Heatmap of changes in differentially expressed genes involved in fatty acid metabolism, amino acid metabolism, and glycogen metabolism (*n* = 3 biological duplicates, 50 male flies for each duplicate), plotted as log10(TPM +1). (C) GSEA analysis of cellular glucan metabolic process treated with IAA in 30-day-old *Drosophil*a (*n* = 3 biological duplicates, 50 male flies for each duplicate). (D) GSEA analysis of cellular lipid catabolic process treated with IAA in 30-day-old *Drosophil*a (*n* = 3 biological duplicates, 50 male flies for each duplicate). (E) GSEA analysis of cellular amino acid catabolic process treated with IAA in 30-day-old *Drosophil*a (*n* = 3 biological duplicates, 50 male flies for each duplicate). (F) GSEA analysis of cellular immune system process treated with IAA in 30-day-old *Drosophil*a (*n* = 3 biological duplicates, 50 male flies for each duplicate). *P* values correspond to the permutation test. *P < 0.05, **P < 0.01 and ***P < 0.001.

**Fig 5:**
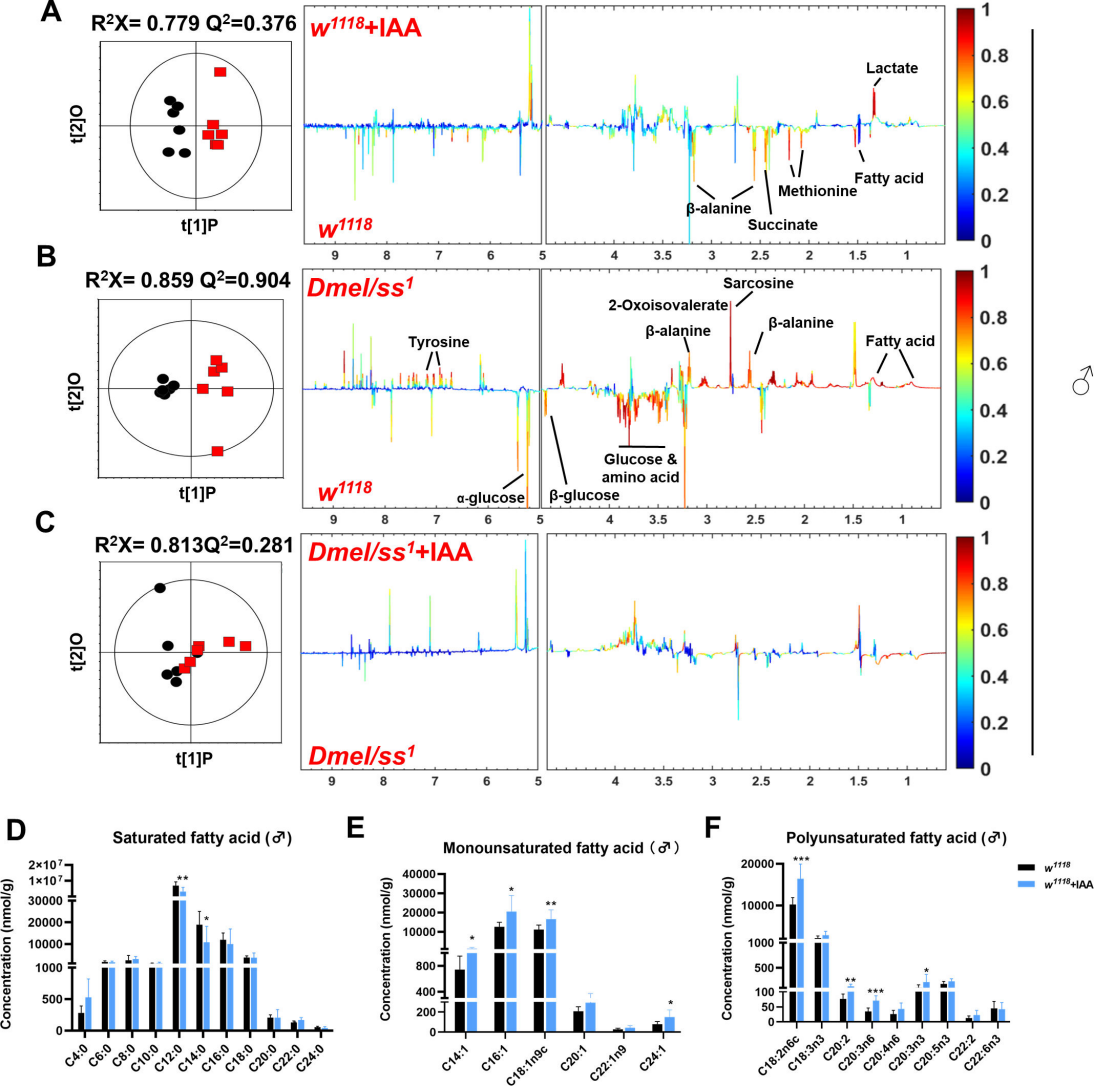
AhR regulates fatty acid metabolism in *Drosophila*. (A–C) NMR-based untargeted metabolomics (*n* = 8 biological duplicates, 50 male flies for each duplicate). OPLS-DA coefficient scores (left) and loading plots (right) from ^1^H NMR spectra of flies in *w^1118^*, *w^1118^* +IAA, *Dmel/ss^1^*, *Dmel/ss^1^* + IAA group (*n* = 6). The color-coded correlation coefficient indicates the significance of the metabolite contribution to the class separation, with a “hot” color (e.g., red) being more significant than a “cold” color (e.g., blue). R^2^X indicates the proportion of variance in the X matrix explained by the model, and Q^2^ reflects the model’s predictive power. (D) Quantification of saturated fatty acids change treated with IAA in 30-day-old *Drosophil*a (*n* = 8 biological duplicates, 10 male flies for each duplicate). *P* values correspond to one-way ANOVA. (E) Quantification of monounsaturated fatty acids change treated with IAA in 30-day-old *Drosophil*a (*n* = 8 biological duplicates, 10 male flies for each duplicate). *P* values correspond to one-way ANOVA. (F) Quantification of polyunsaturated fatty acids change treated with IAA in 30-day-old *Drosophil*a (*n* = 8 biological duplicates, 10 male flies for each duplicate). Data are shown as mean ± SEM. *P* values correspond to one-way ANOVA. *P < 0.05, **P < 0.01, and ***P < 0.001.

Transcriptome analysis revealed that supplementation with IAA significantly increased DNA repair (Fig. 4A) and inhibited the processes in glucan metabolism, lipid catabolism, amino acid catabolism, and immune system (Fig. 4C through F) in *Drosophila*. Genes involved in fatty acid, amino acid, and sugar metabolism were significantly downregulated after supplementation with IAA (Fig. 4B). To verify these metabolic alterations, global NMR-based metabolomics was employed to comparatively screen the metabolic profiling of wild-type and *Ahr* mutant *Drosophila* with and without IAA treatment (Fig. 5A through C; Fig. S5A through C). NMR signal assignments were first performed for metabolites in *Drosophila* using a range of 2D NMR spectra (Table S4). Subsequently, multivariate statistical analysis of the ^1^H NMR data revealed significant reductions in the levels of amino acids (β-alanine and methionine) and succinate in the *w^1118^* strain following IAA supplementation (Fig. 5A; Fig. S5A). Notably, *Ahr* mutation led to a significant elevation in the levels of fatty acids, some amino acids including β-alanine, lysine, sarcosine, 2-oxoisovalerate, and tyrosine (Fig. 5B; Fig. S5B). Interestingly, IAA supplementation induced no significant alterations in these metabolites of *Ahr* mutant flies, especially in male ones (Fig. 5C; Fig. S5C). We further examined the compositional changes in the levels of total fatty acids in aged flies with IAA treatment using targeted GC-MS metabolomics. IAA supplementation induced outstanding upregulation in the levels of monounsaturated fatty acids (MUFAs) (e.g., C14:1, C16:1, and C18:1n9c) and certain polyunsaturated fatty acids (PUFAs) (e.g., C18:2n6c, C20:2, C20:3n6, and C20:3n3) in wild-type *Drosophila* (Fig. 5E and F; Fig. S5E and F).

### SIRT2 is required for AhR activation-mediated longevity in *Drosophila*

**Fig 6:**
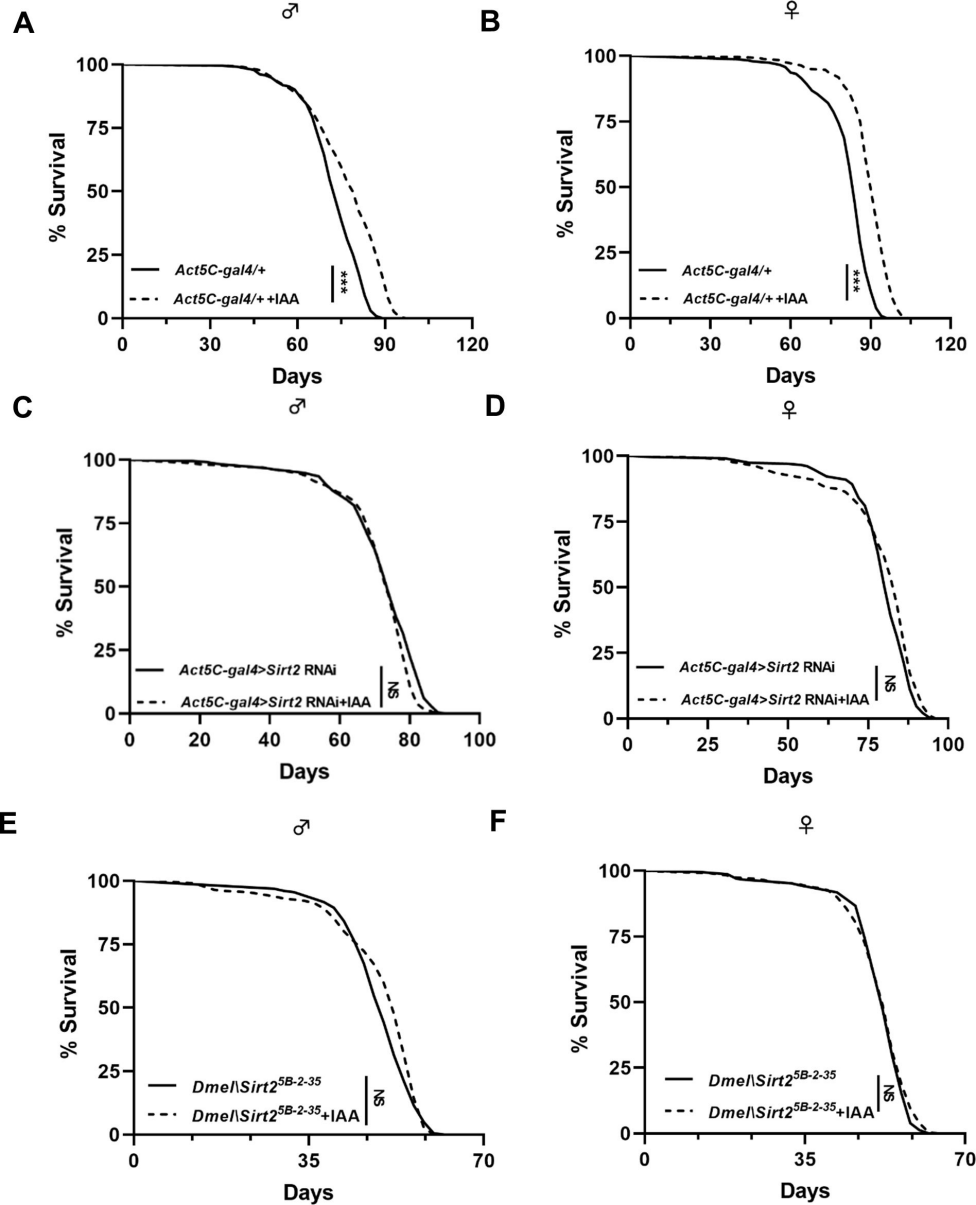
Activation of AhR extends lifespan via Sirt2 in *Drosophila*. (A and B) Kaplan–Meier survival curves of Act5C-gal4/+ flies treated with IAA in both male and female gender (*n* = 5 biological duplicates, 40–45 male flies for each duplicate). (C and D) Kaplan–Meier survival curves of Sirt2 KD (Act5C-gal4/+) flies in both male and female gender (*n* = 5 biological duplicates, 40–45 male flies for each duplicate). (E and F) Kaplan–Meier survival curves of Sirt2 mutant (Sirt2^5B-2-35^) flies in both male and female gender (*n* = 5 biological duplicates, 40–45 male flies for each duplicate). Kaplan–Meier survival curves were analyzed by log-rank (Mantel-Cox) test, *P < 0.05, **P < 0.01, and ***P < 0.001.

Given that AhR activation extends lifespan in *Drosophila*, we next determined the requirement of *Sirt2* in facilitating this aging process using *Sirt2* RNAi and *Sirt2* mutant (Sirt2^5B-2-35^) *Drosophila* with and without IAA treatment. *Act5c-gal4 > Sirt2* RNAi flies and *Sirt2^5B-2-35^* were initially established by *Act5c-gal4* and *Sirt2* RNAi cross and *Sirt2* mutant, respectively. IAA supplementation extended *Act5c-gal4/+* flies (Fig. 6A and B), but both flies with knockdown and mutant of *Sirt2* exhibited no significant changes in the lifespan compared with their corresponding controls (Fig. 6C through F), suggesting that SIRT2 is required for activation of AhR by IAA supplementation-mediated longevity in *Drosophila*.

## DISCUSSION

A high complexity has emerged regarding AhR functions in different diseases with clear discrepancies in tumorigenesis and other metabolic syndrome such as obesity, diabetes, and aging (18). Given its important role in mediating host immunity and metabolic homeostasis involved in pathological and physiological processes, AhR has been implicated in aging and age-related diseases (20). Probably due to its highly active physiological metabolism observed here by NMR-based metabolomics, global *Ahr* mutant *Drosophila* exhibited shortened lifespan, which was consistent with a previous cohort study (23), suggesting that AhR deficiency induced marked prematurely aged phenotypes in mice. Since AhR is a ligand-activated transcription factor, various classes of AhR ligands, including endogenous and exogenous ligands and, indeed, ligands within the same class can differentially modulate AhR activity to induce pathophysiological outcomes. Hence, AhR activity may be a double-edged sword driving both deleterious and beneficial responses in the cellular response process attributed to the AhR ligands and other unknown factors (32). Among various sources for AhR ligands, dietary tryptophan is metabolized by the gut bacteria in the gastrointestinal tract producing indole metabolites, one class of typical AhR ligands, that have been shown to contribute to metabolic disorders. In this study, aged *Drosophila* exhibited strikingly altered microbiota composition, especially downregulated *Lactobacillus*, and reduced AhR ligands including indole, ILA, IAA, and IALD during aging. Similar results were also observed in fecal samples in mice and in individuals of metabolic syndromes, thus making AhR a potential target for anti-aging and improvement of metabolic disorders (18). Actually, supplementation with microbial tryptophan metabolites such as indoles, IAA, and IPA and *Lactobacillus reuteri* strain has been shown to ameliorate the features of some metabolic diseases such as inflammatory bowel diseases (IBDs) and diet-induced obesity by rescuing the impaired AhR activity and regulating gut barrier function (33, 34). Notably, commensal microbiota-derived indole was recently reported to extend the healthspan rather than lifespan in *Caenorhabditis elegans*, *Drosophila,* and mice (22). Interestingly, IAA, one of the indole derivatives acting as an AhR ligand, was identified here to extend the lifespan in *Drosophila* in a dose-dependent manner. IAA treatment also exhibited effective improvement of healthy status in aged *Drosophila*, manifested by marked resistance to starvation and oxidative stress, as well as a significant reduction in gut permeability compared with contemporary controls. These seemingly contradictory results warrant further in-depth investigation.

Mechanistically, AhR activation by IAA supplementation successfully stimulated the expression of SIRT2 at both mRNA and protein levels in *Drosophila*, thereby inhibiting downstream TOR signaling and related fatty acid and amino acid metabolic pathways. It has been shown previously that SIRT2 deficiency exaggerates aging-related cardiac hypertrophy via AMPK inhibition by binding to LKB1, a major upstream kinase of AMPK (29). Another previous study reported that SIRT2 overexpression extended lifespan by inducing the checkpoint kinase BubR1 abundance in male mice (30). Furthermore, caloric restriction (CR) can effectively increase lifespan in flies and rodents with marked increases in NAD^+^ and SIRT2 levels (6, 35, 36). Although these data suggest that SIRT2 exhibits its lifespan extension effect, whether either SIRT2 knockout or mutation can shorten lifespan and the relationship between AhR and SIRT2 during aging remain largely unknown. In the current study, we demonstrate that activated AhR by IAA supplementation binds directly to the *Sirt2* promoter inducing *Sirt2* expression, thus extending lifespan via inhibition of the TOR signaling cascade in aged flies, while both SIRT2 knockdown and mutant flies with IAA supplementation display negligible lifespan extension. These findings strongly suggest that SIRT2 is a key mediator in the AhR activation by IAA treatment-induced longevity in *Drosophila*.

It is well known that reduced TOR signaling is a key mediator at least partly in regulating lifespan extension (7). A recent study showed that dietary restriction (DR), especially dietary amino acid restriction, can extend lifespan by downregulating TORC1 activity in stem niche cells of the fly intestine, thereby maintaining gut homeostasis and ensuring longevity (37). In this study, *Ahr* mutant flies exhibit much higher levels of total fatty acids and amino acids than wild-type flies, which may be one of the reasons why *Ahr* mutation leads to life-shortening. Markedly downregulated fatty acids and amino acid levels in wild-type flies by IAA supplementation that is consistent with inhibition of TORC1 activity further confirmed lifespan extension by IAA supplementation via AhR activation. Interestingly, IAA supplementation markedly elevates the levels of UFAs, thus enhancing the anti-oxidative and anti-inflammatory capabilities that are highly beneficial for the healthy status of flies. Notably, *Ahr* mutant flies treated by IAA exhibit no significant changes in TOR-related metabolic pathways and lifespan compared with their corresponding controls, indicating that AhR is the key mediator in regulating lifespan extension in flies upon IAA supplementation (38,39).

In summary, this study identifies a novel link between AhR and SIRT2 contributing to lifespan in *Drosophila*. Reduced ability of the microbiota to produce AhR ligands from tryptophan such as indole metabolites results in defective gut barrier function and related disruption of metabolic pathways in flies during aging. Supplementation with IAA-producing bacteria (40, 41) or IAA directly markedly extends lifespan via activation of AhR-mediated SIRT2 signaling and subsequent inhibition of TOR-related metabolic pathways (Fig. 6C). These findings indicate that the indole derivative IAA, serving as an endogenous AhR ligand, has the potential to be an anti-aging agent via AhR-mediated SIRT2 signaling cascade in *Drosophila* during aging. AhR-mediated Sirt2 signaling contributing to lifespan extension in flies may be relevant to mammals that warrant further investigation.

## MATERIALS AND METHODS

### *Drosophila* assays

#### Fly stocks and husbandry

*Drosophila* lines used include *w^1118^* #3605, *Dmel\ss^1^* stock #2973, Act5c-gal4 stock #08189, and *Sirt2^5B-2-35^* stock #8839 were obtained from the Bloomington Drosophila Stock Centre (Table S2, Supporting Information). Sirt2 RNAi stock THU0928 was obtained from Tsinghua Fly Center. All flies were reared on standard cornmeal-yeast-agar medium at 25°C with a photoperiod of 12 h:12 h LD (light:dark).

### Lifespan assay

In line with previous protocols (42), after 2–3 days of free mating, flies were separated into male and female and placed in *Drosophila* cages (*n* = 200–230, 40–50 flies per cage for five biological replicates). Different concentrations of IAA were dissolved in DMSO and finally added to the medium at a concentration of 0.25% DMSO. The food was changed every 2 days, and the number of dead flies was counted.

### Body weight assay

Thirty-day-old male and female flies were placed into 1.5 mL EP tubes and weighed using an analytical balance with a precision of 0.01 mg, accounting for the weight of each tube. The weight of each fly was obtained by averaging the net weight of 10 flies in a single tube. Each tube (containing 10 flies) served as a biological replicate. Each treatment typically involved 80 flies (8 tubes, each tube containing 10 flies for 8 biological replicates).

### Food intake assay

After 2–3 days of free mating, flies were separated into male and female for food intake assays. Flies were treated by starvation in an empty tube for 4 hours. Following, they were transferred to a medium containing 2.5% Brilliant Blue for 10 minutes, then quickly frozen and transferred to a centrifuge tube with 300 µL of ddH2O. The samples were then subjected to grinding at 60 Hz for 5 seconds using a homogenizer. After centrifugation, the supernatant (100 µL) was added to the 96-well plate and measured the absorbance at 620 nm. A standard curve was drawn using different concentrations of Brilliant Blue to calculate the food intake. Each treatment typically involved 80 flies (8 tubes, each tube containing 10 flies for 8 biological replicates).

### Starvation tolerance assay

In line with previous protocols (43), for the starvation tolerance experiments, 30-day-old flies with different treatments were transferred in groups of 20–25 flies to tubes containing 1% agar. The number of deceased flies was recorded every 2 hours. Each treatment typically involved 80–100 flies (*n* = 4 biological duplicates, 20–25 flies for each duplicate).

### Oxidative stress assay

For the oxidative stress resistance tests, groups of 20–25 flies from various 30-day treatments were initially starved for 2 hours in tubes containing 1% agar. They were then transferred to filter tubes containing 150 µL of a 5% hydrogen peroxide solution. The number of deceased flies was recorded hourly. Each treatment typically involved 80 flies (8 tubes, each tube containing 10 flies for 8 biological replicates).

### Smurf assay

In line with previous protocols with the Smurf assay (31, 44), flies (*n* = 4 biological duplicates, 20–25 flies for each duplicate) from various 30-day treatments were transferred to a standard medium containing 2.5% (wt/vol) Brilliant Blue. Observations were made at 12, 24, and 48 hour intervals. A fly was considered a “Smurf” if the dye leaked from the gut. The Smurf rating of each fly was evaluated. In the experiment inducing intestinal damage with DSS, the treated flies were transferred to a standard medium containing 3% DSS for 24 hours, followed by the standard medium with Brilliant Blue for the Smurf assay.

### Axenic fly cultures

As per the previous protocol (45), to generate axenic flies, 12 hours of embryos were dechorionated for 2 min in 2.7% bleach (sodium hypochlorite) and washed twice in 75% ethanol for 1 min and then twice with sterile, distilled water. These embryos were transferred into axenic food vials or bottles in a tissue culture hood. The flies were transferred every 1–2 days into new vials with axenic food. To prepare axenic food, plastic vials or bottles were treated by UV for 24 hours and filled with fly food containing the antibiotic cocktail (5 mg/mL ampicillin, 500 mg/mL tetracycline, and 2 mg/mL vancomycin in 50% ethanol) added at 6 mL for each 600 mL of standard fly food. The same concentration of ethanol was added to the standard fly food as the control food.

### Supplementation of *L. plantarum* or *L. reuteri*

Freeze-dried *L. plantarum* or *L. reuteri* powder (1 × 10^10^ cfu/g) was used in this study. For the flies, dried *L. plantarum* or *L. reuteri* was dissolved in PBS and added to the SY food during food preparation at a final concentration of 500 µg/mL. Newly born male *Drosophila* were separated for colonization for 7 days after 3 days of free mating. Total *Drosophila* intestinal DNA was extracted, and a qPCR assay was used to determine the amount of *lactobacillus* in the *Drosophila* intestines.

### Target analysis of tryptophan metabolites

Targeted analyses of tryptophan metabolites in files were performed by multiple-reaction monitoring (MRM) using an ultrahigh-performance liquid chromatograph (Agilent 1290) coupled with a model 6460 triple-quadrupole mass spectrometer (UHPLC-QQQ-MS; Agilent Technologies, Inc.). Procedures of file sample preparation and tryptophan metabolite measurements were in line with previous protocols (46).

### RNA-Seq and transcriptomic data analysis

RNA from ~50 flies was extracted in TRIzol Reagent and purified using TruSeqTM RNA sample prep Kit from Illumina (San Diego, CA). Flies (*n* = 3 biological duplicates, 50 male flies for each duplicate) were considered for the RNA-Seq with each replicate containing purified RNA. Sequencing was performed with the Illumina HiSeq × ten/NovaSeq 6000 sequencer (2 × 150 bp read length) by Shanghai Majorbio Bio-pharm Technology Co., Ltd. For RNA-Seq data analysis, clean reads were mapped to the Drosophila genome database (BDGP6.32). The number of clean reads for each gene was quantified and normalized by Transcripts Per Million mapped reads (TPM), a method that correlates the number of clean reads with transcriptional levels. Differential gene expression analysis was conducted using DESeq2, with genes deemed as differentially expressed (DEGs) upon meeting a threshold of a false discovery rate (FDR) < 0.05 or |log2FC| ≥ 1. KEGG PATHWAY enrichment analysis was performed using KOBAS (http://bioinfo.org/kobas). Multiple testing was conducted using the Benjamini-Hochberg (FDR) method, with a *P*-value threshold of ≤0.05 to define significantly different KEGG pathways. Gene set enrichment analysis (GSEA) was implemented to compare vehicle- and IAA-treated flies using GSEA software (https://www.gsea-msigdb.org/gsea/index.jsp).

### Quantitative real-time PCR

Total RNA was extracted from about 20 mg of flies, 1 μg of RNA was used to synthesize cDNA with All-in-One First-Strand cDNA Synthesis SuperMix for qPCR (One-Step gDNA Removal) kit (TransScript). Using the SYBR master mix, quantitative real-time PCR (qPCR) was conducted with a real-time PCR system (ABI StepOne; Applied Biosystems Co., Ltd., China). qPCR conditions were set to 40 cycles of 95°C for 20 s, 95°C for 30 s, and 60°C for 30 s with Actin5C as a reference. The ΔΔCT method, where CT is the threshold cycle, was used in the reaction analysis. The primers of genes used for QPCR are shown in Table S3 (Supporting Information).

### Western blot analysis

For protein analysis, flies were lysed in radioimmunoprecipitation assays (RIPA), supplemented with a cocktail of protease and phosphatase inhibitors. The protein concentration was ascertained using a bicinchoninic acid (BCA) protein assay kit (Beyotime Biotechnology). Proteins were separated by 10% SDS-PAGE electrophoresis and transferred to polyvinylidene difluoride (PVDF) membranes. After a rapid blockade, the membranes were incubated with primary antibodies (including those against β-tubulin, SIRT2, S6k, and phospho-S6K) and secondary antibodies (goat anti-rabbit lgG-HRP). The blots were then probed using enhanced chemiluminescence (ECL) HRP substrate (Millipore) and visualized with a ChemiDoc imaging system (Bio-Rad). The relative levels of proteins were quantified against β-tubulin levels.

### Chromatin immunoprecipitation

ChIP-PCR/qPCR assay was conducted by BeyoChIP Enzymatic ChIP Assay Kit (Protein A/G magnetic beads) according to the manufacturer’s instructions (Cell Signaling Technology, #9005). In brief, 30 mg flies were fixed in 1% formaldehyde for 10 min at 37°C and then quenched with 125 mM glycine for 10 min. Fixed tissues were lysed for 10 min on ice and added micrococcal nuclease (MNase) to generate 200–800 bp fragments. Generated DNA fragments were used as input control. Immunoprecipitations were performed with anti-AhR and rabbit IgG. The immune complexes were collected by incubation with protein A/G magnetic beads, and the beads were washed with low salt, high salt, and ChIP buffer. The samples were then reverse cross-linked at 65°C by adding Tris-EDTA with 1% SDS. After proteinase K digestion, DNA was purified with a PCR/DNA purification kit (Beyotime Biotechnology) for quantitative real-time PCR analysis by specific primers.

### AhR-specific short-hairpin RNA

In line with previous protocols (47), following 2 hours of starvation treatment in serum-free DMEM, HEK 293T cells were transfected with either a non-targeting control (shCtr) or AhR-specific (shAhR) shRNA for a duration of 4–6 hours using the Lipofectamine 3000 transfection reagent (Invitrogen). The culture medium was subsequently switched to DMEM supplemented with 10% fetal bovine serum. After 48–72 hours, cells were selected using 1 mg/mL and maintained with 500 ng/mL puromycin. The knockdown of AhR was verified via reverse transcription polymerase chain reaction (PCR) and Western blot (WB).

### Luciferase reporter assay

The promoter region of the Sirt2 gene (from position −2000 to +10) was inserted into the firefly luciferase vector pGL4-basic. 293T cells were transfected with a control vector (pGL4-basic) or vector containing the Sirt2 promoter (pGL4-Sirt2-pro), together with the renilla luciferase vector pRL-TK as an internal control. The cells were also co-transfected with empty vector (Plko.1-NC) or vector expressing shAhR (Plko.1-AhR) by Lipofectamine 3000 Transfection Reagent (Thermo Fisher Scientific). Sirt2 promoter activity was measured by a dual-luciferase assay system (Beyotime) and results were normalized against the activity of the pRL-TK control group.

### Gut microbiota analysis

Gut microbiota composition was analyzed by 16S rRNA gene sequencing. In the *Drosophila* study, the flies were rinsed with PBS and placed into EP tubes after removing the heads of the flies and sterilizing the surface with 75% alcohol. The total DNA was extracted from the flies (*n* = 8 biological duplicates, about 30 flies for each duplicate). The 16S rRNA gene PCR process used 338F 806R primers. The 16S rRNA gene amplicon sequence library was established as per the protocol of 16S Metagenomic Sequencing Library Preparation (Illumina, United States). Paired-end sequencing (2 × 300 bp) was executed using an Illumina MiSeq platform provided by Shanghai Majorbio Bio-pharm Technology Co., Ltd. Functional predictions were derived from 16S rRNA sequences employing PICRUSt2 and subsequently annotated with the KEGG (Kyoto Encyclopedia of Genes and Genomes) database. Additional details regarding the preparation of the 16S rRNA gene amplicon sequence library, statistical analysis, and data manipulation are available in the supplemental materials.

### NMR-based metabolomics

^1^H-NMR experiments were conducted as previously described. Briefly, the one-dimensional proton spectra of *Drosophila* samples were acquired at 298K using a Bruker Avance III 600 MHz nuclear magnetic resonance (NMR) spectrometer (Bruker BioSpin, Germany), equipped with a Bruker inverse cryogenic probe. A standard NOESY pulse sequence (recycle delay−90°−*t*_1_−90°−*t*_*m*_−90°−acquisition) was applied to the fly extracts, with parameters set as follows: recycle delay time of 2.0 s, *t*_1_ of 3.0 μs, and mixing time (*t*_*m*_) of 80 ms. The 90° pulse length was about 13.46 μs, and 64 transients were collected into 32 K data points for each spectrum, within a spectral width of 20 ppm. Two-dimensional (2D) ^1^H−^1^H COSY, ^1^H−^1^H TOCSY, ^1^H−^13^C HSQC, and ^1^H−^13^C HMBC NMR spectra of flies were used for NMR signal identification (Table S4). Detailed information about sample extraction and data analysis was provided in supplemental materials.

### Fatty acid analysis

In line with previous protocols (46), a targeted analysis of long-chain fatty acids (LCFAs) in whole flies was performed using a Shimadzu 2010 Plus gas chromatograph (GC) MS, equipped with a flame ionization detector (FID) and a CP-FFAP CB capillary GC column (10 m by 0.1 mm, 0.1 mm; Agilent Technology). C17:0 was utilized as an internal standard for the quantification of LCFAs. The preparation of samples and procedures for fatty acid measurements are provided in the supplemental materials.

### Statistical analysis

All Kaplan–Meier lifespan curves were analyzed by log-rank (Mantel–Cox) test. R software (4.2.1) and GraphPad Prism software (GraphPad 8.0.2) were used for statistical data analysis and graphical illustrations. All data plotted by column are shown as means ± SD. For two-group comparisons: unpaired t test was used when data met the criteria for parametric analysis (normal distribution assessed by Shapiro–Wilk normality test or nonparametric analysis). For more than two groups of comparison, one-way or two-way ANOVA with the Bonferroni post hoc test was performed. The specific analysis methods were clarified in the figure legends. *P* < 0.05 was set as the statistical significance.

## Data Availability

The 16S rRNA gene sequencing data (PRJNA1026831) and RNA-seq data (PRJNA1026761) have been deposited in the National Center for Biotechnology Information GenBank repository. All data generated or analyzed during this study are available from the corresponding author upon reasonable request.

## SUPPLEMENTAL MATERIAL

Fig. S1 msystems.01665-24-s0001.tif Altered microbiota composition and reduced AhR agonists in *Drosophila* during aging.

Fig. S2 msystems.01665-24-s0002.tif Altered microbiota composition and reduced AhR agonists during aging in female *Drosophila*.

Fig. S3 msystems.01665-24-s0003.tiff Colonized *Lactobacillus* increases the levels of IAA in *Drosophila*.

Fig. S4 msystems.01665-24-s0004.tiff Supplement of IAA extends lifespan and improves healthspan in female *Drosophila*.

Fig. S5 msystems.01665-24-s0005.tiff AhR regulates fatty acid metabolism in female *Drosophila*.

Supplemental text msystems.01665-24-s0006.docx Additional experimental details.

Legends msystems.01665-24-s0007.docx Supplemental figure legends.

Supplemental tables msystems.01665-24-s0008.docx Tables S1 to S4.

ASM does not own the copyrights to Supplemental Material that may be linked to, or accessed through, an article. The authors have granted ASM a non-exclusive, world-wide license to publish the Supplemental Material files. Please contact the corresponding author directly for reuse.
