# Supplementary figures and images for: Microbiota-derived indole acetic acid extends lifespan through the AhR-Sirt2 pathway in Drosophila
Source: mSystems. 2025 Apr 8;10(5):e01665-24. doi: 10.1128/msystems.01665-24 (PMC12090787; doi:10.1128/msystems.01665-24)

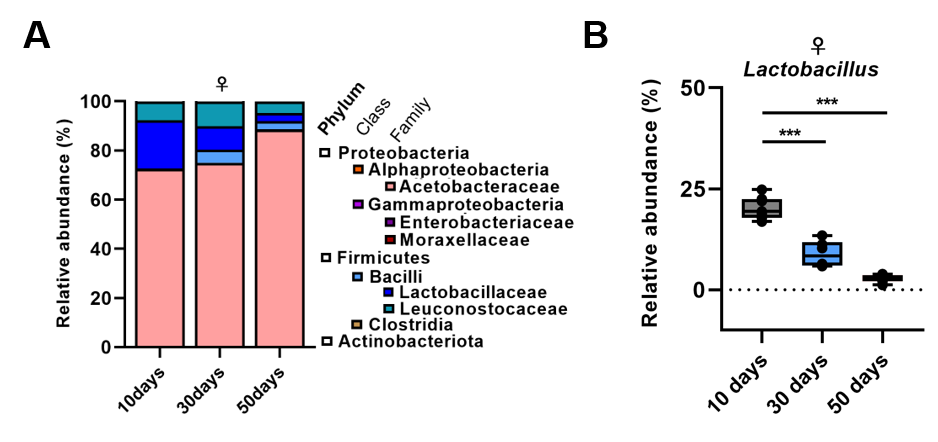

Supplement: Fig. S1 — Altered microbiota composition and reduced AhR agonists in Drosophila during aging. [file msystems.01665-24-s0001.tif]

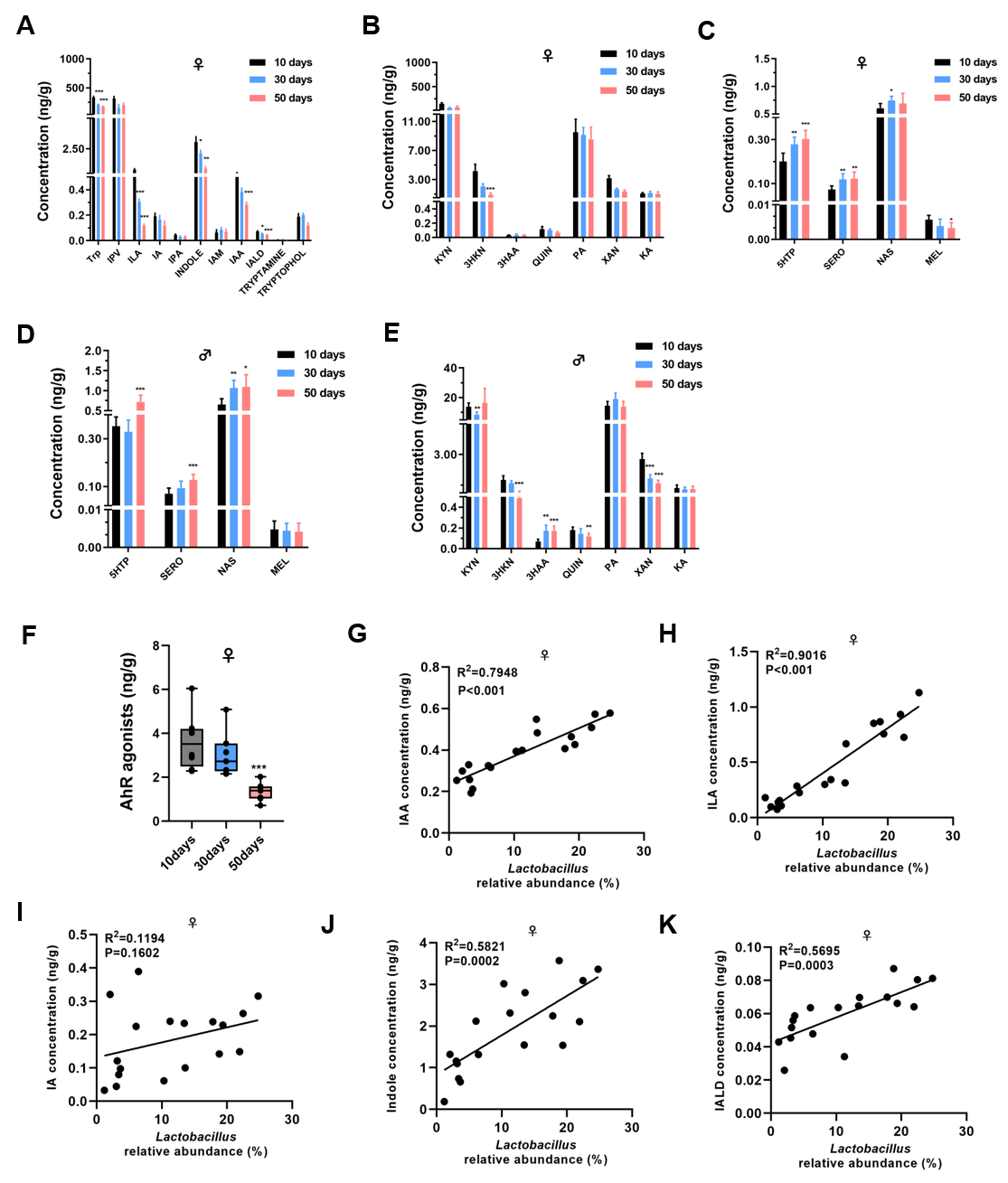

Supplement: Fig. S2 — Altered microbiota composition and reduced AhR agonists during aging in female Drosophila. [file msystems.01665-24-s0002.tif]

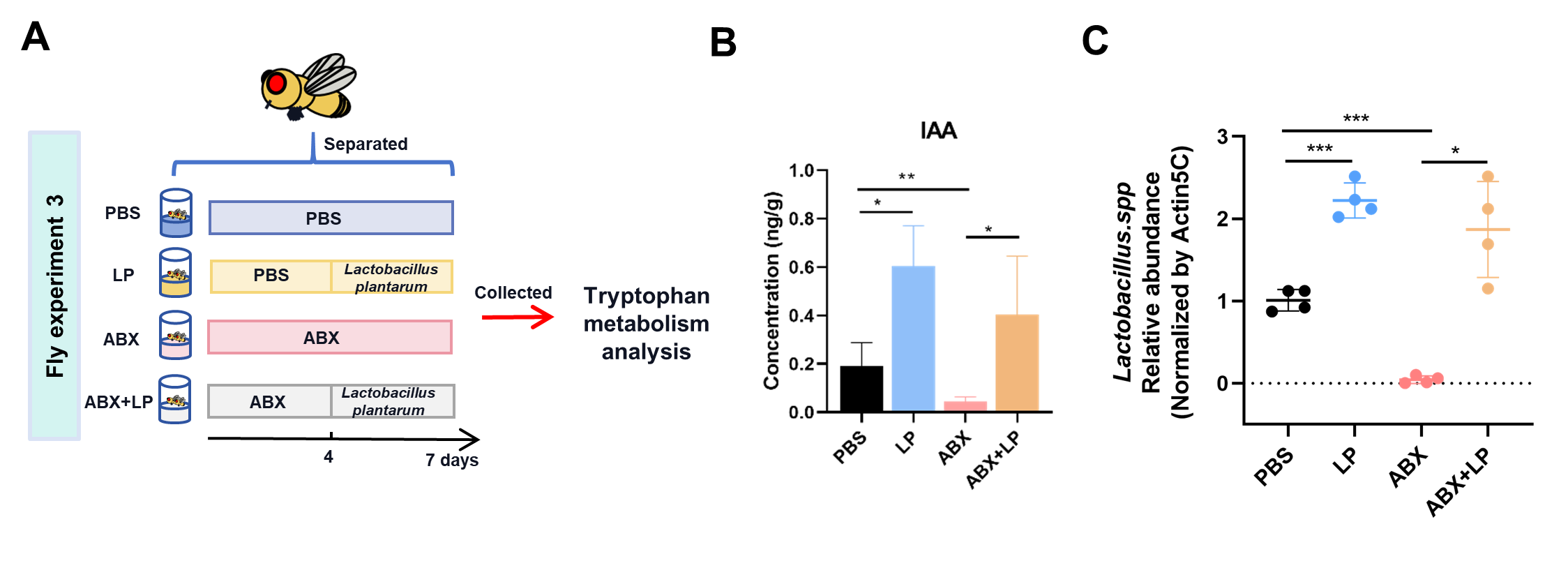

Supplement: Fig. S3 — Colonized Lactobacillus increases the levels of IAA in Drosophila. [file msystems.01665-24-s0003.tiff]

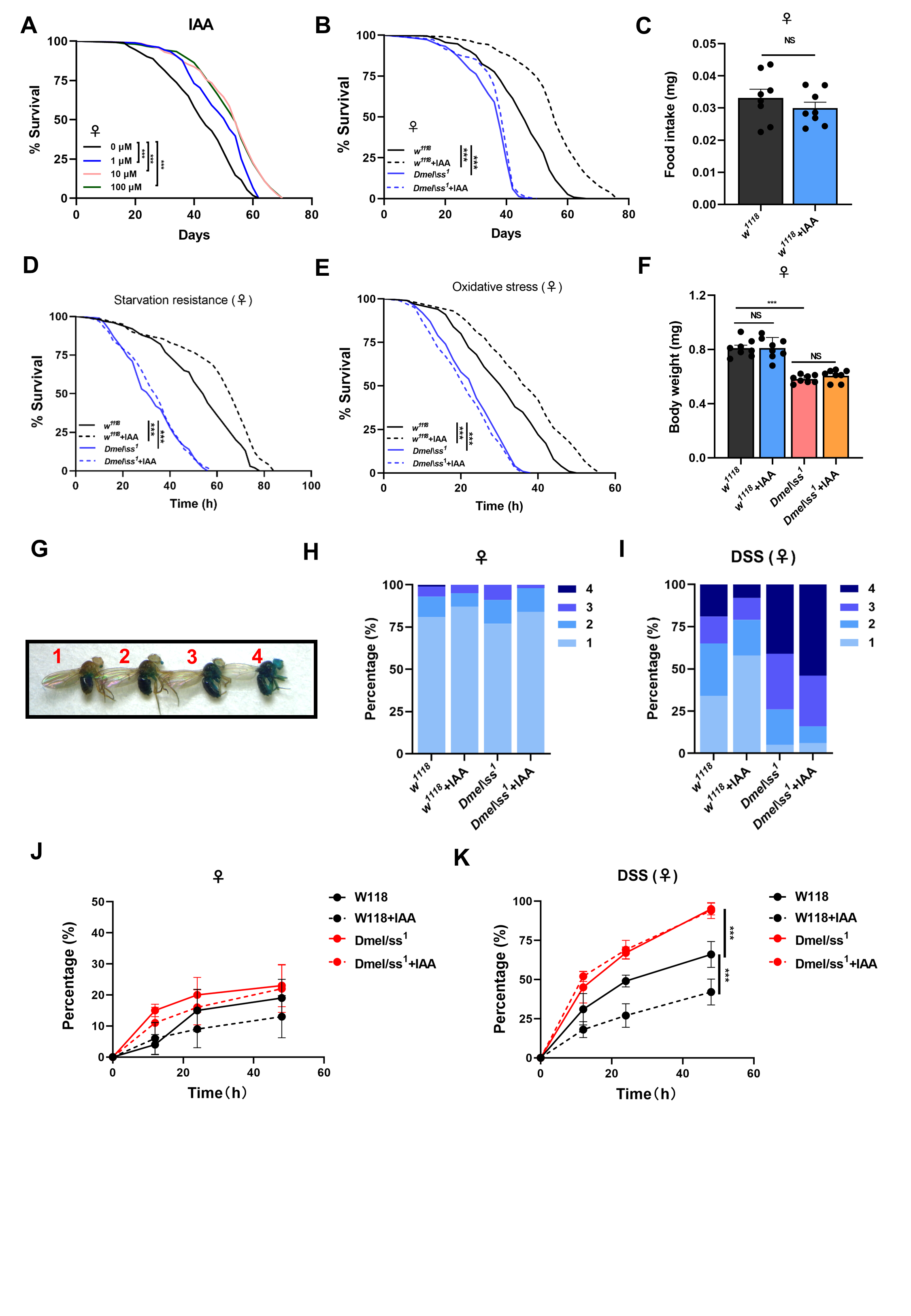

Supplement: Fig. S4 — Supplement of IAA extends lifespan and improves healthspan in female Drosophila. [file msystems.01665-24-s0004.tiff]

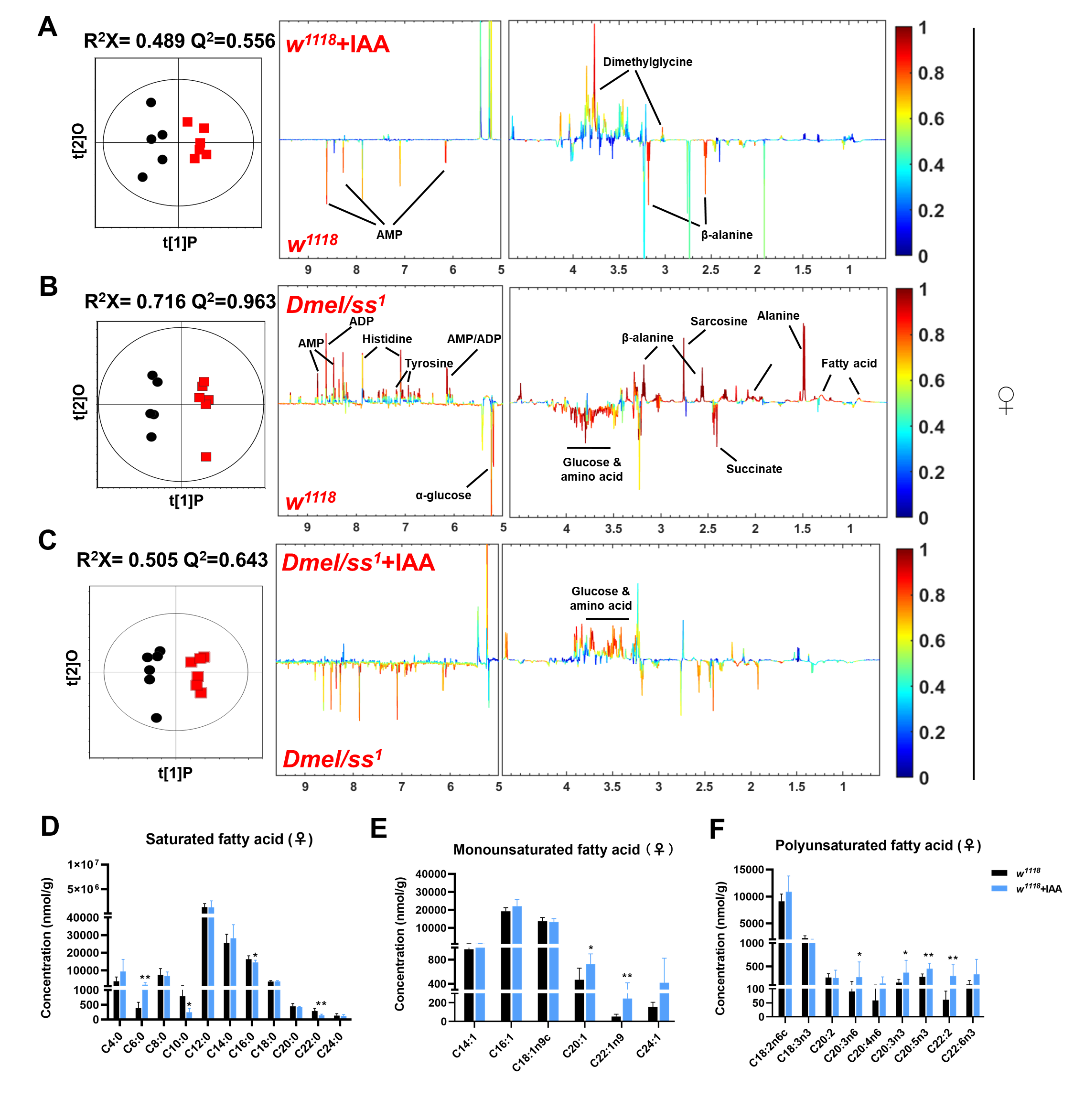

Supplement: Fig. S5 — AhR regulates fatty acid metabolism in female Drosophila. [file msystems.01665-24-s0005.tiff]
