# Supplementary material for: Microbiota-derived indole acetic acid extends lifespan through the AhR-Sirt2 pathway in Drosophila
Source: mSystems. 2025 Apr 8;10(5):e01665-24. doi: 10.1128/msystems.01665-24 (PMC12090787; doi:10.1128/msystems.01665-24)
Supplement: Supplemental text — Additional experimental details. [file msystems.01665-24-s0006.docx]

**Supplemental Materials and methods**

**Extraction of tryptophan metabolites and UPLC-QQQ detection.** Tryptophan metabolites extraction was performed from hole flies (~10 pcs). Samples mixed with internal standard (10 μL d5-TRP) were respectively homogenized with 400 μL cooled methanol and 50 μL acetonitrile: water solution (1:1 v/v) containing 0.1% formic acid using the Qiagen Tissue-Lyser (Retsch GmBH, Germany) at 20 Hz for 90 s. After extraction for two times, the combined supernatants were collected and evaporated into dryness following centrifugation. Serum sample (10 μL) was uniformly mixed with 10 μL of internal standard (d5-trp), 150 μL cooled methanol and 10 μL acetonitrile: water solution (1:1 v/v) containing 0.1% formic acid. After centrifugation for 20 min (4 °C), the supernatants were collected and lyophilized for removing methanol in vacuum. Dried extracts were reconstituted in 100 μL of acetonitrile: water solution (1:1 v/v) containing 0.1% formic acid. Qualitative and quantitative analyses of tryptophan metabolites were performed using an ultrahigh performance liquid chromatography (Agilent 1290) coupled with a 6460 triple quadrupole mass spectrometry (UHPLC-QQQ-MS, Agilent Technologies, Inc.). The precursor ions of tryptophan metabolites were pre-scanned through multiple reaction monitoring (MRM) of all sample mixtures and the structures were identified through MS/MS spectra. Quantification of tryptophan metabolites was performed using calibration curves based on MRM and the ratios of the integrated peak areas of tryptophan metabolites and internal standards.

**Gut microbiota analysis.**

For 16S rRNA gene sequencing analysis, total DNA of flies (~30 pcs) was extracted using the E.Z.N.A.® soil DNA Kit (Omega Bio-tek, Norcross, GA, U.S.), according to the manufacturer’s protocol. The 16S rRNA gene amplicon sequence library was prepared as described in the protocol of 16S Metagenomic Sequencing Library Preparation (Illumina, United States). Briefly, the V3-V4 region of 16S rRNA gene was ampliﬁed using a KAPA HiFi Hot Start PCR Kit (KAPA Biosystem, USA). Dual-index barcodes were added to the amplicon target by the index PCR using a Nextera® Index Kit (Illumina, USA). Amplicons were puriﬁed using AMPure XP beads and quantiﬁed using a KAPA library quantitative kit (KAPA Biosystem, USA). Equimolar amounts of puriﬁed amplicons were pooled and paired-end sequencing (2 × 300 bp) were performed using an Illumina MiSeq platform by Shanghai Majorbio Bio-pharm Technology Co., Ltd. After demultiplexing, the resulting sequences were merged with FLASH (v1.2.11) and quality filtered with fastp (0.19.6). Then the high-quality sequences were de-noised using Deblur plugin in the Qiime2 (version 2021.11) pipeline with recommended parameters, which obtained single nucleotide resolution based on error profiles within samples. Deblur denoised sequences are usually called amplicon sequence variants (ASVs). In this study, these denoised sequences were assigned to bacterial features, which are synonymous to ASVs. To minimize the effects of sequencing depth on alpha and beta diversity measure, the number of sequences from each sample was rarefied to 20000, which still yielded an average Good’s coverage of 97.90%. The taxonomy of these features was performed using the Vsearch consensus taxonomy classifier implemented in Qiime2 and the SILVA 16S rRNA database (version 138). Most statistical analysis and data manipulation were conducted with vegan, reshape2, and ggplot2 packages using R software.

**NMR-Based Metabolomics**

About 25 flies were extracted three times with cooled methanol/water extraction solution (2/1, v/v) using the Qiagen Tissue-Lyser (Retsch GmBH, Germany) at 20 Hz for 90 s. After collection of all three extracts, the combined supernatants were lyophilized for removing methanol by vacuum Freeze Drier. The obtained powder was reconstituted in 600 μL phosphate buffer (0.1 M, K2HPO4:NaH2PO4 = 4:1, pH ≈ 7.4) containing 100% D2O and 0.001% TSP as an internal reference. Typical one-dimensional 1H NMR spectra were acquired for liver extracts at 298 K on a Bruker Avance III 600 MHz spectrometer (Bruker BioSpin, Germany) equipped with a Bruker inverse detection cryogenic probe. Standard NOESY pulse sequence (recycle delay-90°-t1-90°-tm-90°-acquisition) was used for liver extracts with parameters as recycle delay time of 2.0 s, t1 of 3.0 μs, and mixing time (tm) of 80 ms.

For NMR signals identification, several two-dimensional (2D) NMR spectra including 1H−1H COSY, 1H−1H TOCSY, 1H−13C HSQC, and 1H−13C HMBC were recorded for flies samples. All 1H NMR spectra phase and baseline were manually corrected and referenced internally to the TSP peak at 0.00 ppm for flies’ extracts using Topspin 3.6 (V3.1, Bruker Biospin, Germany). The spectra region δ 0.5-δ 9.6 ppm with removal of water signals were integrated with an equal width of 0.002 ppm (1.2 Hz) for all the samples using the MestRenova 9.0. After integration, the wet weight normalization method was performed for each bucketed region of flies’ extracts. Multivariate data statistical analysis was conducted using SIMCA-P+ 13.0 (Umetrics, Sweden). In brief, principal component analysis (PCA) was firstly used to check the information on group separation and potential outliers. Orthogonal projection to latent structures with discriminant analysis (OPLS-DA) was subsequently employed using NMR data scaled to unit variance. All the models were further assessed by a 7-fold cross-validation with CV-ANOVA (p < 0.05). For extracting and interpreting the results of OPLS-DA model, the color-coded loading plots were conducted to extract or discriminant the significantly changed metabolites after back-transformation of the integrated NMR data using MATLAB script (V7.8, MA).

**Fatty acids compositional analysis**

Collected flies were rapidly mixed with 100 μL chilled methanol/10mg flies (HPLC grade) and subjected to freeze-thaw with liquid nitrogen. After vortexing, the mixture was homogenized using a Visualizer (Qiagen, Germany). 100 μL cells homogenate and 20 μL internal standards (1 mg/mL C17:0 and 0.5 mg/mL C23:0) were mixed with 1 mL MeOH/hexane solution (4:1, v/v). The methylation and quantification of fatty acids was performed as previously described with some improvement. In brief, acetyl chloride (100 μL) was used to trigger the methylation reaction. The mixture was kept in dark at 25 °C for 24 h and terminated with K2CO3 solution on ice. A total of 200 μL hexane was used to extract the methylated fatty acids and the procedure was repeated for three times. Following centrifugation, supernatants of lipid phase fraction were collected and then evaporated to dryness at room temperature in a rotating evaporator. The methylated fatty acids were dissolved in hexane (50 μL) and then analyzed by GC-FID/MS spectrometer (Shimadzu Scientific Instruments, Japan) equipped with DB-225 column (10 m, 0.1 μm thickness, Agilent) and an ion flame detector. The programmed conditions were set as follows: kept the temperature at 55 °C for 5 min and then increased to 205 °C at rate of 30 °C min, kept at 205 °C for 3 min later then increased to 230 °C, and then kept at 230 °C for further 5 min. Each fatty acid was quantified with the FID data from its signal integrals and internal standards, and corrected by Relative Response Factor (RRF). The chromatogram of fatty acids and method performance of the GC-MS based targeted metabolomics were provided in the supplement.
