# Supplementary material for: Microbiota-derived indole acetic acid extends lifespan through the AhR-Sirt2 pathway in Drosophila
Source: mSystems. 2025 Apr 8;10(5):e01665-24. doi: 10.1128/msystems.01665-24 (PMC12090787; doi:10.1128/msystems.01665-24)
Supplement: Legends — Supplemental figure legends. [file msystems.01665-24-s0007.docx]

**SUPPLEMENTAL MATERIAL FIGURE LEGENDS AND TABLE CAPTIONS**

**Figure S1. Altered microbiota composition and reduced AhR agonists in *Drosophila* during aging.** (A) Metabolites involved in KYN metabolism pathway change during aging in male *Drosophila* (n = 8 biological duplicates, 10 male flies for each duplicate). (B) Metabolites involved in 5-HT metabolism pathway change during aging in male *Drosophila* (n = 8 biological duplicates, 10 male flies for each duplicate). Pvalue were obtained by one-way ANOVA with Bonferroni post hoc test, **p* < 0.05, ***p* < 0.01 and ****p* < 0.001.

**Figure S2. Altered microbiota composition and reduced AhR agonists during aging in female *Drosophila*.** (A) Changes of gut microbiota in 10, 30, 50 days-old female *Drosophila* (n = 6-8 biological duplicates, 10 female flies for each duplicate). (B) Changes of *Lactobacillus* abundance in 10, 30, 50 days-old female *Drosophila* (n = 6-8 biological duplicates, 10 female flies for each duplicate). (C) Metabolites involved in Indole metabolism pathway (n = 8 biological duplicates, 10 female flies for each duplicate). (D) Metabolites involved in KYN metabolism pathway (n = 8 biological duplicates, 10 female flies for each duplicate). (E) Metabolites involved in 5-HT metabolism pathway (n = 8 biological duplicates, 10 female flies for each duplicate). (F) Changes in the content of AhR ligands (n = 8 biological duplicates, 10 female flies for each duplicate). (G-K) Correlation between *Lactobacillus* and AhR agonists. Pvalue were obtained by one-way ANOVA with Bonferroni post hoc test, **p* < 0.05, ***p* < 0.01 and ****p* < 0.001.

**Figure S3. Colonized *Lactobacillus* Increase the Levels of IAA in *Drosophila*. (A)** Schematic illustration of the fly experiment. (B) IAA levels in WT, germ-free *Drosophila* and colonized with *Lactobacillus plantarum* (n = 6 biological duplicates, 10 male flies for each duplicate)*.* (C) Detection of *Lactobacillus* levels in *Drosophila* by qPCR (n = 4 biological duplicates, 10 male flies for each duplicate). Pvalue were obtained by one-way ANOVA with Bonferroni post hoc test, **p* < 0.05, ***p* < 0.01 and ****p* < 0.001.

**Figure S4. Supplement of IAA Extends Lifespan and Improves Healthspan in female *Drosophila*.** (A) Kaplan–Meier lifespan curves of supplement IAA with different concentrations in *w^1118^* flies (n = 5 biological duplicates, 40-45 female flies for each duplicate). (B) Kaplan–Meier lifespan curves of supplement with 50 μM IAA in *w^1118^* and *AhR* mutant flies (n = 5 biological duplicates, 40-45 female flies for each duplicate). (C) Food intake assay of supplement with 50 μM IAA in *W^1118^* flies (n=8 biological duplicates, 10 female flies for each duplicate). (D) Starvation tolerance of supplement with 50 μM IAA in *w^1118^* and *AhR* mutant flies (n = 8 biological duplicates, 40-45 female flies for each duplicate). (E) Oxidative stress of supplement with 50 μM IAA in *w^1118^* and *AhR* mutant flies (n = 8 biological duplicates, 40-45 female flies for each duplicate). (F) Body weight in 30 days-old *w^1118^* and *AhR* mutant flies (n=8 biological duplicates, 10 female flies for each duplicate). (G) Smurf rating (n = 4 biological duplicates, 20-25 female flies for each duplicate). (H, J) Smurf rating of 30-days male flies in *w^1118^* and *AhR* mutant flies of supplement with 50 μM IAA. (I, K) Smurf rating of 30-days female flies after 3 % DSS treatment in *w^1118^* and *AhR* mutant flies of supplement with 50 μM IAA. Data are shown as mean ± SEM. Pvalue were obtained by one-way ANOVA with Bonferroni post hoc test. Kaplan–Meier lifespan curves were analyzed by log-rank (Mantel–Cox) test, **p* < 0.05, ***p* < 0.01 and ****p* < 0.001.

**Figure S5. AhR Regulates Fatty Acid Metabolism in Female *Drosophila*.** (A-C) NMR-based untargeted metabolomics (n = 8 biological duplicates, 50 female flies for each duplicate). OPLS-DA coefficient scores (left) and loading plots (right) from 1H NMR spectra of flies in *w^1118^*, *w^1118^*+IAA, Dmel/ss^1^, Dmel/ss^1^+IAA group (n = 6). (D-F) Quantification of saturated fatty acids, monounsaturated fatty acids and polyunsaturated fatty acids (n = 8). Data are shown as mean ± SEM. Pvalue were obtained by one-way ANOVA with Bonferroni post hoc test, **p* < 0.05, ***p* < 0.01 and ****p* < 0.001.
