# Supplementary material for: Microbiota-derived indole acetic acid extends lifespan through the AhR-Sirt2 pathway in Drosophila
Source: mSystems. 2025 Apr 8;10(5):e01665-24. doi: 10.1128/msystems.01665-24 (PMC12090787; doi:10.1128/msystems.01665-24)
Supplement: Supplemental tables — Tables S1 to S4. [file msystems.01665-24-s0008.docx]

**Table S1. Summary of the lifespan experiments.**

| Figures | Conditions | Maximum lifespan ± SEM | Median lifespan ± SEM | N | Censored | *P* value |
| --- | --- | --- | --- | --- | --- | --- |
| Figure 2B | 0 μmoL  1 μmoL  10 μmoL  100 μmoL | 64.4 ± 1  66.4 ± 0.4  71.6 ± 0.4  69.6 ± 0.4 | 49.2 ± 1.1  54 ± 0.7  55.8 ± 1  55.2 ± 1.5 | 221  207  210  217 | 4  13  7  8 | < 0.001  < 0.001  < 0.001 |
| Figure 2C | *W^1118^*  *W^1118^* + IAA  *Dmel\ss^1^*  *Dmel\ss^1^* + IAA | 60.4 ± 0.7  68.8 ± 1  39.2 ± 0.8  39.6 ± 0.7 | 42.6 ± 1.3  49.2 ± 1.2  32.8 ± 0.5  33.2 ± 0.8 | 220  216  224  214 | 1  4  0  5 | < 0.001  < 0.001 |
| Figure 2E | *W^1118^*  *W^1118^* + IAA  *Dmel\ss^1^*  *Dmel\ss^1^* + IAA | 54 ± 0.8  61 ± 1  42.5 ± 1  43 ± 1.3 | 32.5 ± 2.8  41.2 ± 2.7  28 ± 1.4  29 ± 0.6 | 100  99  100  100 | 0  1  0  0 | < 0.001  < 0.001 |
| Figure 2F | *W^1118^*  *W^1118^* + IAA  *Dmel\ss^1^*  *Dmel\ss^1^* + IAA | 33 ± 0.6  38 ± 0.8  22 ± 0.8  20 ± 0.8 | 22 ± 0  25 ± 0.6  13 ± 1  13.5 ± 1.3 | 100  100  99  100 | 0  0  1  0 | < 0.001  < 0.001 |
| Figure S4A | 0 μmoL  1 μmoL  10 μmoL  100 μmoL | 59.6 ± 0.4  62 ± 0.6  66.8 ± 0.5  70.4 ± 0.4 | 45 ± 1.1  52 ± 0.7  54.8 ± 1  55 ± 1.5 | 210  210  209  213 | 10  5  10  5 | < 0.001  < 0.001  < 0.001 |
| Figure S4B | *W^1118^*  *W^1118^* + IAA  *Dmel\ss^1^*  *Dmel\ss^1^* + IAA | 62 ± 1.1  78.8 ± 0.5  45.2 ± 0.8  46.4 ± 1 | 46.8 ± 1.5  57 ± 0.6  38 ± 0  39 ± 0.4 | 226  216  217  218 | 0  4  3  2 | < 0.001  < 0.001 |
|  |  |  |  |  |  |  |
| Figure S4D | *W^1118^*  *W^1118^* + IAA  *Dmel\ss^1^*  *Dmel\ss^1^* + IAA | 75 ± 1.3  82 ± 2  53.5 ± 1.5  52 ± 2.6 | 54.5 ± 2.1  67 ± 2.4  32 ± 2.4  34.5 ± 2.1 | 98  99  100  101 | 1  2  0  0 | < 0.001  < 0.001 |
| Figure S4E | *W^1118^*  *W^1118^* + IAA  *Dmel\ss^1^*  *Dmel\ss^1^* + IAA | 47.5 ± 1.3  56 ± 0.8  36 ± 0.8  35.5 ± 0.5 | 32 ± 1.2  37 ± 1.7  23.5 ± 0.5  22.5 ± 0.5 | 100  100  100  98 | 0  0  0  1 | < 0.001  < 0.001 |
| Figure 6A | *Act5C-gal4/+*  *Act5C-gal4/*+ IAA | 89±0.9  97±1.1 | 73±0.9  79±1.1 | 232  227 | 0  0 | < 0.001 |
| Figure 6B | *Act5C-gal4/+*  *Act5C-gal4/*+ IAA | 96±0.9  104±1.1 | 84±0.9  90±1.1 | 234  244 | 0  0 | < 0.001 |
| Figure 6C | *Act5C-gal4 > Sirt2* RNAi  *Act5C-gal4 > Sirt2* RNAi + IAA | 86.8 ± 1.4  85.6 ± 0.7 | 74.4 ± 1.9  74 ± 0.6 | 235  229 | 0  1 | > 0.05 |
| Figure 6D | *Act5C-gal4 > Sirt2* RNAi  *Act5C-gal4 > Sirt2* RNAi + IAA | 92.8 ± 1  94.8 ± 0.5 | 80.8 ± 0.8  83.2 ± 1.3 | 229  231 | 1  3 | > 0.05 |
| Figure 6E | *Dmel\Sirt2^5B-2-35^*  *Dmel\Sirt2^5B-2-35^* + IAA | 59.6 ± 0.7  59.2 ± 0.5 | 50 ± 1.3  52 ± 0.6 | 225  230 | 1  2 | > 0.05 |
| Figure 6F | *Dmel\Sirt2^5B-2-35^*  *Dmel\Sirt2^5B-2-35^* + IAA | 60.4 ± 0.7  61.2 ± 1 | 53 ± 0.8  52.8 ± 1.4 | 231  226 | 4  0 | > 0.05 |

**Table S2. Fly stocks information**

| Primer | Source | Stock |
| --- | --- | --- |
| w^1118^ | Bloomington Drosophila Stock Centre | #3605 |
| Dmel/ss^1^ | Bloomington Drosophila Stock Centre | #2973 |
| Sirt2^5B-2-35^ | Bloomington Drosophila Stock Centre | #8839 |
| Act5C-gal4 | Bloomington Drosophila Stock Centre | #81890 |
| Sirt2 RNAi | Tsinghua Fly Center | THU0928 |

**Table S3. Sequence of primers.**

| Primer | Sequence |
| --- | --- |
| Actin5C-F | TTGTCTGGGCAAGAGGATCAG |
| Actin5C-R | ACCACTCGCACTTGCACTTTC |
| Sirt2-F | CTGGCTAATACCGCGAGCTT |
| Sirt2-R | TTGGCGGTTTGCTGCTTTTT |
| Sirt2 promotor-F | AGCGGAGCACCCGATAATTT |
| Sirt2 promotor-R | GAAACCATGCACACGCCAAT |

**Table S4. Nuclear magnetic resonance metabolite assignment in *Drosophila*.**

| Number | Metabolites | Groups | δ1H (ppm) |
| --- | --- | --- | --- |
| 1 | Lactate (Lac) | αCH  βCH3 | 4.12(q)  1.33(d) |
| 2 | Alanine (Ala) | αCH  βCH3 | 3.78(q)  1.48(d) |
| 3 | Lysine (Lys) | αCH  εCH2  βCH  γCH2  δCH2 | 3.76(t)  3.03(t)  1.92(m)  1.72(m)  1.45(m) |
| 4 | Acetate (Acet) | CH3 | 1.92(s) |
| 5 | Pyruvate (Pyr) | CH3 | 2.37(s) |
| 6 | Succinate (Succ) | CH2 | 2.41(s) |
| 7 | Taurine (Tau) | CH2SO3  CH2NH2 | 3.27(t)  3.42(t) |
| 8 | Creatine (Cr) | CH3  CH2 | 3.03(s)  3.93(s) |
| 9 | Glucose (Glc) | 1-CH  2-CH  3-CH  4-CH  5-CH  6-CH2 | 5.24(d)  3.54(dd)  3.73(dd)  3.49(m)  3.83(m)  3.83(dd) |
| 10 | Glycogen | 1-CH  2-CH | 5.41(d)  3.63(dd) |
| 11 | Fumarate | CH | 6.52(s) |
| 12 | Tyrosine (Tyr) | βCH2  αCH  3 or 5-CH  2 or 6-CH | 3.06, 3.14(dd)  3.94(t)  6.90(d)  7.20(d) |
| 13 | Histidine (His) | CH  CH | 7.09(s)  7.89(s) |
| 14 | Formate | CH | 8.46(s) |
| 15 | Inosine | 2-H  8-H  2-H′(ribose)  4-H′(ribose)  3-H′(ribose)  5-H′(ribose)  CH2 | 8.35(s)  8.24 (s)  6.10(t)  4.77(t)  4.44(m)  4.29(m)  3.87(dd),  3.99(dd) |
| 16 | Adenosine monophosphate (AMP) | 2-CH  7-CH  2'-CH | 8.61(s)  8.27(s)  6.15(d) |
| 17 | Unsaturated  fatty acids | R-CH3  R-CH3  R-CH2  CH2C=C  CH=CH  C=CCH2C=C  CH2CH2COO  CH2COO | 0.88(t)  0.89(t)  1.29(m)  2.01(m)  5.30(m)  2.75(m)  1.59(m)  2.24(m) |
| 18 | Methyl phosphate | OCH3 | 3.47(d) |
| 19 | β-glucose (β-Glc) | 1-CH  2-CH  3-CH  4-CH  5-CH  6-CH  6′-CH | 4.65(d)  3.25(dd)  3.50(t)  3.40(dd)  3.47(m)  3.73(dd)  3.90(dd) |
| 20 | citrate | CH2  CH2’ | 2.55(d)  2.68(d) |
| 21 | fumarate | CH | 6.53(s) |
| 22 | guanosine | CH of guanine | 8.01(s) |
| 23 | NAD^c^ | 8-CH of adenine  5-CH of nicotinamide  2-CH of adenine  4-CH of nicotinamide  6-CH of nicotinamide  2-CH of nicotinamide | 8.18(s)  8.19(m)  8.43(s)  8.83(dd)  9.14(m)  9.35(s) |
| 24 | fatty acid | CH3  (CH2) n  -CH=CH- | 0.89(m)  1.27(m)  5.30(m) |
| 25 | PEG^c^ | CH2 | 3.72(s) |
| 26 | β-alanine | αCH2  βCH2 | 2.56(t)  3.19(t) |
| 27 | Methionine (Met) | CH3  βCH2  γCH2  αCH | 2.14(s)  2.20(m)  2.65(t)  3.90(m) |
| 28 | Methyl phosphate | OCH3 | 3.47(d) |
| 29 | Glutathione (GSH) | Glu α  Glu β  Glu γ  Cys α  Cys β | 3.78(t)  2.16(m)  2.56(m)  4.57(dd)  2.94(m) |
| 30 | Sarcosine | CH3 | 2.74(s) |
| 31 | Dimethylamine (DMA) | CH3 | 2.72(s) |
| 32 | Adenosine diphosphate (ADP) | 2-CH  7-CH  2’-CH | 8.54(d)  8.27(d)  6.15(d) |
| 33 | 2-Oxoisovalerate | CH3  CH | 1.12(d)  3.02(m) |
